# An algorithmic multiple attribute decision-making method for heart problem analysis under neutrosophic hypersoft expert set with fuzzy parameterized degree-based setting

**DOI:** 10.7717/peerj-cs.1607

**Published:** 2023-12-04

**Authors:** Muhammad Ihsan, Muhammad Saeed, Agaeb Mahal Alanzi, Hamiden El-Wahed Khalifa

**Affiliations:** 1 Department of Mathematics, University of Management & Technology, Lahore, Pakistan; 2 Department of Mathematics, College of Science and Arts, Qassim University, Al-Badaya, Saudi Arabia; 3 Department of Operations and Management Research, Faculty of Graduate Studies for Statistical Research, Cairo University, Giza, Egypt

**Keywords:** Fuzzy set, Fuzzy soft expert set, Hypersoft expert set, Optimization algorithm, Decision-making

## Abstract

A fuzzy parameterized neutrosophic hypersoft expert set (FpNHse-set) is one of the family members of fuzzy parameterized structures and a valuable extension of the neutrosophic soft expert set as well as the neutrosophic hypersoft set. This structure involves a multi-argument approximate function that has the ability to change the sub-characteristic pairs in the form of power set of universe. The main function of this structure is the classification of each character into sub-characteristic valued sets. Due to this prominent property, this mathematical structure is useful for uncertainties and also helps make the decision-making process more adaptable and dependable. By using the algebraic and basic ideas of the FpNHse-sets, a useful strategy, especially for medical diagnosis, known as Sanchez’s method has been used in this study. To see a reformed process for the medical diagnosis of heart disease, a useful combination of FpNHse-set and a modified Sanchez’s method has been made in this context. By using the real data from the Cleveland data set for heart disease, the implementation of the reform process has been made to see its veracity. Finally, a clear comparison of the used study with its existing studies has been made for the purpose of benefits.

## Introduction

In different scientific directions, there are many mathematical techniques like the fuzzy set (𝔽-set) (Zadeh, 1965) and the intuitionistic fuzzy set (*IF*-set) (Atanassov & Atanassov, 1999) for the solution of difficult situations having various types of uncertainties. The fuzzy set-like technique is for the truth value of an item belonging to a certain description space, and the second is for both the truth and false values of an item with the property of dependency on each other. Both of these techniques fail when there is an involvement of neutral value in an item that also has previous two values. To overcome such laziness, a new technique called neutrosophic set (ℕ-set) (Smarandache, 2006) has been brought about with the situation of dependency upon each other. The three techniques described above are all hindered in some way by handling issues involving uncertainties. The limitation of the parameterizations method may be the cause of these obstacles. To address these issues, a mathematical tool that is devoid of all such obstacles is required. A new technique of soft set 𝕊-set has been introduced (Molodtsov, 1999), which allows for the determination of this meagerness. The 𝕊-set’s features, operations, laws, relations, and functions with applications in decision-making were researched by different analysts like Maji, Biswas & Roy (2003) and Ali et al. (2009). To make the combination of 𝕊-set and three techniques *i.e.*, (𝔽-set) (Zadeh, 1965), (*IF*-set) (Atanassov & Atanassov, 1999), (ℕ-set) (Smarandache, 2006) respectively, called fuzzy soft set (𝔽s-set), intuitionistic fuzzy soft set (𝕀Fs-set), and neutrosophic soft set (ℕs-set), a new parameterizations technique has been suggested for their hybridised structures. The 𝕊-set like structures have certain limitations regarding the multi-decisive opinions. In order to address these limitations, Alkhazaleh & Salleh (2011) (2007) developed the “soft expert set (𝕊e-set), which combines a 𝕊-set and an expert set. They organized the basic findings and firmly emphasized the challenges with decision-making. Ihsan, Saeed & Rahman (2021) made a useful extension to the 𝕊e-set by introducing convexity in this environment. By creating a lovely extension in the 𝕊-set employing a fuzzy environment, Alkhazaleh & Salleh (2014) initiated the concept of the fuzzy soft expert set (𝔽se-set). Ihsan et al. (2021) also studied some fundamental properties of convexity in a 𝔽se-set environment. They created its workings and applied them to problem-solving situations. By using examples, Broumi & Smarandache (2015) successfully made the combination 𝕊e-set and intuitionistic fuzzy set and named it an intuitionistic fuzzy soft expert set (𝕀Fse-set) for the discussion of truth and falsity values with multiple definitive opinions and how it may be used in multi-criteria decision-making issues. Ihsan et al. (2023) embedded convexity in this structure of 𝕀Fse-set while discussing its valuable results. The 𝕊-set like structures are insufficient to handle such sets in many real-world scenarios where unique attributes must be categorised into parametric non-overlapping groupings. The problem of categorization of parameters into parametric non-overlapping groupings is handled by considering the structures of hypersoft set (ℍs-set) (Smarandache, 2018). The main function of this structure is to convert the function of single argument into a function of multi-arguments. The ℍs-fundamental set’s characteristics, aggregation procedures, laws, relations, and functions have been studied by researchers (Abbas, Murtaza & Smarandache, 2020; Saeed et al., 2022) for correct comprehension and future use in a variety of domains. In a complex set context, Rahman et al. (2020) made the combination of fuzzy, intuitionistic fuzzy, neutrosophic sets and hypersoft sets. Rahman, Saeed & Smarandache (2020) embedded convexity in the ℍs-set context. To see the role of hypersoft set like structures in decision-making, Debnath (2021a) made use of fuzzy hypersoft set (𝔽Hs-set) by utilising its weight operator. The intuitionistic fuzzy hypersoft set (𝕀FHs-set) was created by Yolcu, Smarandache & Öztürk (2021) and its numerous operations were covered. Saqlain et al. (2019) looked into the accuracy function, similarity measurements, and some of the main useful characteristics of the neutrosophic set like structures called neutrosophic hypersoft set (ℕHs-sets). Using the accuracy function, they used decision-making strategies like TOPSIS to address issues in the actual world. Saeed et al. (2021a) and Saeed et al. (2021b) first introduced the idea of mappings, which is now employed in managerial for medicating analysis. Many researched faced problems of knowing the multi-decisive opinions while using a single structure. Then Ihsan, Rahman & Saeed (2021b) made useful extension in the structure of hypersoft set by introducing the experts opinions and formed a new structure called hypersoft expert set (ℍse-set). Ihsan, Rahman & Saeed (2021a) and Ihsan, Saeed & Rahman (2022) developed fuzzy hypersoft expert set (𝔽Hse-set) and neutrosophic hypersoft expert set (ℕHse-set). All the abbreviation used in the introduction section shown in Table 1.

### Research gap, motivation and novelty

To assess the research need, novelty, and purpose of the proposed study, consider the following factors:

1. Kirisci, Demir & Simsek (2021) presented an algorithm that can select patients at risk of developing heart disease based on cardiovascular data using 𝕊-set. A medical case was examined as a real-life application to see if the proposed method is applicable. Das et al. (2021) identified the most important diseases that are deadly as compared to other diseases by using Rough Set taken from soft computing technique, and then applied the same approach to identify the most important factor that causes heart diseases, before using Time Series to project the ailments and demonstrating their exponential growth rate. Kirisci (2020a) presented a strategy to make health choices that was connected to Celik-Yamak’s fuzzy soft set using Sanchez’s method and made use of the Cleveland data set. Muthukumar & Krishnan (2016) talked about some of the fundamental characteristics of 𝕀Fs-sets and presented a new similarity measure for them. The suggested similarity score has also been used to demonstrate medical diagnosing issues in a fictitious investigation. To identify and keep track of patients with heart failure, Abdel-Basset et al. (2020) brought up a novel system based on IoT and computer-supported diagnosis. The suggested health care system made it possible to make diagnoses with greater accuracy despite ambiguous data. They recommended the use of a neutrosophic multi-criteria decision-making technique to help patients and doctors determine whether a patient has heart failure. Kirisci (2019) used the Riesz summability technique for heart disease and medical decision making purposes. Al-Sharqi, Ahmad & Al-Quran (2023) suggested a new technique based fuzzy parameterized interval complex neutrosophic soft set and applied to medical diagnoses especially in heart diseases. Hassan et al. (2017) developed a new fuzzy soft expert system to check the coronary artery disease with different parameters. Long, Meesad & Unger (2015) suggested a new method for the heart disease based on interval type-2 fuzzy logic system. As a decision support system for the diagnosis of cardiac disease, they suggested a very useful method. Sanz et al. (2014) introduced a new method for heart disease analysis by combing fuzzy set classification with interval valued fuzzy set based. Their method remained good due to high classification rate. Kumar, Inbarani & Azar (2015) developed a brand-new automatic classification system for use in taking decisions and heart rhythm evaluation. A back propagation neural network and a bijective soft set have been combined to create the described sorting technique. Lashari, Ibrahim & Senan (2017) developed a novel algorithm based on fuzzy soft set for the heart disease analysis.
2. The 𝔽se-set, 𝕀Fse-set, ℕse-set, and other techniques have been widely used to handle decision-making issues in a variety of contexts. However, under a number of circumstances, these structures show shortcomings in classifying the elements similar to their parameterized levels. Especially, it can be inferred that each parameter set element’s parameterized degree is taken as one in the existing literature. The introduction of the fuzzy parameterized aspect was one of the key turning points in the evolution of soft sets, soft expert sets, and their generalisations. In particular, when combined with the more precise generalisations of soft and soft expert sets like 𝔽se-set, 𝕀Fse-set, and other hybrid models mentioned above, this new aspect has further improved the theories of soft and 𝕊s-sets and made them better suited to be used in handling managerial difficulties. First, Cagman & Enginoglu (2011) presented the idea of a fuzzy parameterized soft set (FP-SS) and assigned a level of priority to each parameter in the set, establishing the fuzzy parameterized aspect. Bashir & Salleh (2012) introduced the concept of fuzzy parameterized soft expert sets(FP-SES). This element was then attached to the soft sets, soft expert sets, and fuzzy sets generalisations that already existed. As a generalisation of the work by Hazaymeh et al. (2012) introduced the concept of fuzzy parameterized fuzzy soft expert sets (FP-FSES). Selvachandran & Salleh (2016) introduced the concept of fuzzy parameterized intuitionistic fuzzy soft expert set (FP-IFSES) in their article. These sets, however, are limited in their ability to deal with inconsistent and imprecise information, which is typically present in real-world circumstances. The fuzzy parameterized single valued neutrosophic soft expert set (FP-SVNSES), developed by Al-Quran & Hassan (2016) to address this weakness, outperforms these models with three independent membership functions. Rahman et al. (2022a) converted into neutrosophic hypersoft set with fuzzy parameterizations settings. The fuzzy parameterized aspect of the FP-SVNSES model gives it additional advantages than single valued neutrosophic soft expert set in that it delivers more information, improving the quality of the information that single valued neutrosophic soft expert set presents, which in turn improves the accuracy of the final decision. The goal of the fuzzy parameterization notion is to give each characteristic (or sub-attribute) in the context of an approximate function with just one argument (or multiple arguments) a fuzzy grade. Using models that resemble soft sets, various scholars like (Yılmaz & Eraslan, 2012; Kirisci, 2020b), and have thoroughly investigated this concept. These models treat fuzzy subsets as items in their codomain and fuzzy parameters as elements inside the domain of soft approximate mapping. Other scholars have more recently expanded the idea of fuzzy parameterization to matrices in a soft set context (Rahman et al., 2022b; Rahman et al., 2022c). They have introduced and characterized a variety of new matrix-based features and operations and used them in decision-making, geographical study, and arranging of data that is numerical.
3. In contrast, there are many instances in real-world observations where the parameters are insufficient to allow for the proper decision-making and necessitate classification into the appropriate sets based on parametric values. By using the structure of hypersoft set having the function of multi-argument to handle these arrangements, such issues have been addressed.
4. The ℕHse-set is a type of structure which covers of the fuzzy, intuitionistic fuzzy, neutrosophic-sets, and their hypersoft sets with expert sets. The ℕHse-set is created to address the certain limitations of the above described structures. For example, the 𝔽se and  𝕀Fse-structures ignore the property of function having multi-argument and degree of indeterminacy, whereas the ℕHse-set ignores both these properties.
5. The considered structure, FpNHse-set is a peculiar design that not only generalises the current structures but also enhances them by utilising the function multi-argument. To express how uncertain the neutrosophic numbers of the FpNHse-set are, a parameterized degree is assigned to them. In this sense, handling unknown data with caution is a more flexible and all-encompassing strategy.

The main contributions of this study are summarised in the following sentences:

1. The FpNHse-fundamental set’s concepts and algebraic operations are described by the help of illustrative numerical.
2. Contrary to prior access, the important values of attributes (sub-attributes) taking from the Cleveland data set are investigated first in terms of their operative and lingual responsibilities, and then these important values are transformed into parameterized values with the help of applicable procedure.
3. The FpNHse-set environment modifies Sanchez’s method, a traditional access to medicinal analysis, in order to create a connection between the decision-makers (medical specialists), the patients who are being observed, and the prescribed qualities.
4. The Cleveland data set’s necessary real data, the modified Sanchez’s technique, and the FpNHse-sets concept are combined to create a reform process that is suggested for the medical identification of cardiac issues.
5. Implementing the reform process in a scenario based on real-world problems allows for the validity of the approach to be evaluated.
6. In order to assess the reliability, flexibility, and advantageous aspects of the proposed approach, two types of comparison are needed: structural comparison and computation-based comparison. The first one is meant to assess the flexibility of the proposed structure, whereas the second one is meant to check the reliability of the presented approach.

## Preliminaries

To make sure that readers can understand the planned study, this section of written work introduces some basic concepts and definitions by reading the relevant literature. In this part, set will be expressed by $\hat {\hbar }$ and $\mathcal{Z}$ as a universe of discourse and set of experts is by $\mathcal{X}$ and ℕ will be a set of opinions, ${\mathcal{C}}_{1}=\hat {\hbar }\times \mathcal{X}\times \mathbb{N}$. While $P(\mathcal{Z})$ will be used as a power set.

**Table 1:** List of abbreviations used in the article.

Full name	Abbreviations	Full name	Abbreviations
Fuzzy set	𝔽-set	Hypersoft set	ℍs-set
Intuitionistic Fuzzy	*IF*-set	Fuzzy hypersoft set	𝔽Hs-set
Neutrosophic set	ℕ-set	Intuitionistic Fuzzy hypersoft set	𝕀FHs-set
Soft set	𝕊-set	Neutrosophic hypersoft set	ℕHs-set
Fuzzy soft set	𝔽s-set	Hypersoft expert set	ℍse-set
Intuitionistic fuzzy soft set	𝕀Fs-set	Fuzzy hypersoft expert set	𝔽Hse-set
Neutrosophic soft set	ℕs-set	Neutrosophic hypersoft expert set	ℕHs-set
Hypersoft set	ℍs-set	Fuzzy hypersoft set	𝔽Hs-set
Soft expert set	𝕊e-set	Fuzzy parameterized soft set	FP-SS
Fuzzy Soft expert set	𝔽e-set	Fuzzy parameterized soft expert set	FP-SES
Intuitionistic Fuzzy Soft expert set	𝕀Fe-set	Fuzzy parameterized Fuzzy soft set	FP-FSS
Neutrosophic soft expert set	ℕse-set	Fuzzy Pa. Intuitionistic fuzzy expert set	FP-IFSES
Fuzzy Parameterized single valued Neutrosophic soft expert set	FP-SVNSES	Fuzzy Parameterized Neutrosophic hypersoft expert set	FpNHse-set

Definition 2.1 (Ihsan, Rahman & Saeed, 2021b) An ℍSe-set Υ_*HSe*_ is defined by as ${\mathrm{&Upsi;}}_{HSe}:\mathrm{&Lambda;}&rarr; P(\mathcal{Z})$ where Λ⊆𝒞 = 𝒫 × 𝒳 × ℕ and $\mathcal{P}={\ddot{\Omega }}_{1}\times {\ddot{\Omega }}_{2}\times {\ddot{\Omega }}_{3}\times ...\times {\ddot{\Omega }}_{k}$, while ${\ddot{\Omega }}_{i}$, *i* = 1,2,3, …,k show the different characteristic graded sets corresponding to k different parameters ℵ_1_, ℵ_2_, ℵ_3_, …, ℵ_*k*_.

Definition 2.2 (Ihsan, Rahman & Saeed, 2021a) An ℍSe-set (Υ_*HSe*_, Λ) is named as a fuzzy ℍSe-set, intuitionistic fuzzy ℍSe-set, neutrosophic ℍSe-set, when $P(\mathcal{Z})$ is removed and in this place new things like $F(\mathcal{Z})$, $IF(\mathcal{Z})$, $N(\mathcal{Z})$ are used and these are all the subsets of $\mathcal{Z}$ representing the collection of fuzzy, intuitionistic fuzzy and neutrosophic sets.

Definition 2.3 (Al-Quran & Hassan, 2016) A pair $({\mathrm{&Xi;}}_{F},\mathcal{C})$ is called a FPsvNse-set on $\mathcal{Z}$, such that ${\mathrm{&Xi;}}_{F}:{\mathcal{C}}_{1}&rarr; FP(\mathcal{Z})$ and $FP(\mathcal{Z})$ represents a collection of single-valued neutrosophic subsets of $\mathcal{Z}$.

## Fuzzy parameterized neutrosophic hypersoft expert set and set-theoretic operations

Here, the definition of FpNHse-set and some basic operations are provided together with numerical examples. The real-world scenario, which necessitates the establishment of the FpNHse-set, is covered first. It is a frequent observation that a panel is assembled in any recruitment procedure to interview the initial inspected applicants. This panel typically includes a chairperson and several members with knowledge of the subject. All panel members are instructed to evaluate each candidate’s aptitude and appropriateness for the open positions by taking into account predetermined assessing parameters and their sub-parametric values expressed as sets. They are also told to use their professional judgement in three dimensions when evaluating candidates for multi-argument tuples, *i.e.,* to suggest, reject, or remain neutral. The chairperson has the power to rank the specialist’s view points of the decision-makers in terms of their approach of approval. Three situations in this scenario must be handled by one model, briefly:

1. The circumstance that indicates the fundamental grouping of the properties into their associated sub-characteristic grades as various sets.
2. It is necessary that the function of multi argument be able to handle the domain having a multi-argument function in which tuples are sub-parametric values in order for it to function.
3. The condition that needs decision-makers to present their judgement as neutrosophic values, which guarantee the opinions three components truth, neutral value, and real non-membership.
4. The situation that makes it necessary for the parameterized degree to be reflected in order to gauge the level of judgement for the things beneath discussion.

The currently available study is insufficient to offer any numerical structure that would address all of the aforementioned circumstances collectively in one structure. Our deficiency serves as the inspiration for this study. All of the aforementioned cases can be managed as a single structure using the suggested structure, the FpNHse-set. The FpNHse-set is made up of three components: (i) the fuzzy parameterized degree-based context (ii), neutrosophic context, and (iii) hypersoft context. The FpNHse context is necessary in a wide range of different real-world situations, including choosing products, diagnosing illnesses, choosing projects, analysing risks, etc.

Definition 3.1 The structure FpNHse-set Υ_*F*_ can be defined as ${\Upsilon }_{F}=\{ ((\zeta /{\curlyvee }_{F}(\zeta ),{\ddot{S}}_{i},{\ddot{G}}_{i}),\eta /{\Phi }_{F}(\eta ));\forall \zeta \in \mathcal{P},{\ddot{S}}_{i}\in \mathcal{X},{\ddot{G}}_{i}\in \mathcal{N}\} $, with ${&cuvee; }_{F}:\mathcal{C}&rarr; FP(\mathcal{Z})$, Φ_*F*_ is an approximate function of FpNHse-sets such that ${\mathrm{&Phi;}}_{F}:\mathcal{C}&rarr; NP(\mathcal{Z})$.

Example 3.2 Consider a scenario in which the medical director of a public hospital appoints a group of cardiologists to evaluate heart conditions by keeping track of the proper characteristics and their pertinent sub-characteristics grades for the purpose of study. A chairperson leads the committee and is in charge of making the final choice. The chairperson has the authority to carefully examine the received viewpoints in accordance with their acceptability level. Other committee members will contribute their professional (expert) opinions as decision-makers. The set of discourse $\{ {\widehat{\mathbf{P}}}_{1},{\widehat{\mathbf{P}}}_{2},{\widehat{\mathbf{P}}}_{3},{\widehat{\mathbf{P}}}_{4}\} $ includes four sorts of cardiac problems (alternatives) that are taken into consideration. The committee members reached agreement on the parameters **c**_1_ = chest pain type, **c**_2_ = resting blood pressure (mmHg), and **c**_3_ = serum cholesterol (mg/dL), before setting them. After careful examination, the characteristics are further divided into their respective related parametric-valued sets, **J**_1_ = {**c**_11_ = *typicalangina*, **c**_11_ = *atypicalangina*}, **J**_2_ = {**c**_21_ = 150, **c**_22_ = 180}, and **J**_3_ = {**c**_31_ = 320}. The cartesian product of the parameters with experts and their opinions is $\mathbf{J}\times \widehat{\mathbf{E}}\times \widehat{\mathbf{O}}=\{ ({\zeta }_{1},{\zeta }_{2},{\zeta }_{3},{\zeta }_{4})\} $ to get the parametric pairs of characteristics. The committee persons are instructed to submit their comments in neutrosophic parts for every issue, while bearing in mind the preference of the parametric tuples. As functions of multi-argument of the FpNHse-set, the members’ expert opinions are gathered along with the parameterized degree the monitor assigned for the side of acceptance of the opinions obtained. These elements are listed below: 

\begin{eqnarray*}\Lambda ({\zeta }_{1}/0.2,{\hat {\Xi }}_{1},1)= \left\{ \begin{array}{@{}l@{}} \displaystyle ({\hat {P}}_{1}/\prec 0.5,0.3,0.4\succ ),({\hat {P}}_{2}/\prec 0.6,0.2,0.5\succ ),\\ \displaystyle ({\hat {P}}_{3}/\prec 0.3,0.4,0.4\succ ),({\hat {P}}_{4}/\prec 0.3,0.7,0.2\succ ) \end{array} \right\} , \end{eqnarray*}

\begin{eqnarray*}\Lambda ({\zeta }_{2}/0.3,{\hat {\Xi }}_{2},0)= \left\{ \begin{array}{@{}l@{}} \displaystyle ({\hat {P}}_{1}/\prec 0.4,0.5,0.2\succ ),({\hat {P}}_{2}/\prec 0.2,0.7,0.3\succ ),\\ \displaystyle ({\hat {P}}_{3}/\prec 0.6,0.3,0.5\succ ),({\hat {P}}_{4}/\prec 0.4,0.6,0.5\succ ) \end{array} \right\} , \end{eqnarray*}

\begin{eqnarray*}\Lambda ({\zeta }_{3}/0.4,{\hat {\Xi }}_{1},0)= \left\{ \begin{array}{@{}l@{}} \displaystyle ({\hat {P}}_{1}/\prec 0.7,0.8,0.3\succ ),({\hat {P}}_{2}/\prec 0.9,0.2,0.6\succ ),\\ \displaystyle ({\hat {P}}_{3}/\prec 0.3,0.7,0.6\succ ),({\hat {P}}_{4}/\prec 0.8,0.1,0.2\succ ) \end{array} \right\} , \end{eqnarray*}

\begin{eqnarray*}\Lambda ({\zeta }_{4}/0.5,{\hat {\Xi }}_{2},1)= \left\{ \begin{array}{@{}l@{}} \displaystyle ({\hat {P}}_{1}/\prec 0.3,0.7,0.9\succ ),({\hat {P}}_{2}/\prec 0.4,0.8,0.5\succ ),\\ \displaystyle ({\hat {P}}_{3}/\prec 0.7,0.4,0.5\succ ),({\hat {P}}_{4}/\prec 0.6,0.7,0.3\succ ) \end{array} \right\} . \end{eqnarray*}

The FpNHse-set can be described as $(,\widetilde {\mathrm{S}})=$

\begin{eqnarray*} \left\{ \begin{array}{@{}l@{}} \displaystyle \left( ({\zeta }_{1}/0.2,{\widehat{\Xi }}_{1},1),\Lambda ({\zeta }_{1}/0.2,{\widehat{\Xi }}_{1},1)= \left\{ \begin{array}{@{}l@{}} \displaystyle ({\widehat{P}}_{1}/\prec 0.5,0.3,0.4\succ ),({\widehat{P}}_{2}/\prec 0.6,0.2,0.5\succ ),\\ \displaystyle ({\widehat{P}}_{3}/\prec 0.3,0.4,0.4\succ ),({\widehat{P}}_{4}/\prec 0.1,0.7,0.2\succ ) \end{array} \right\} \right) ,\\ \displaystyle \left( ({\zeta }_{2}/0.3,{\widehat{\Xi }}_{2},0),\Lambda ({\zeta }_{2}/0.3,{\widehat{\Xi }}_{2},0)= \left\{ \begin{array}{@{}l@{}} \displaystyle ({\widehat{P}}_{1}/\prec 0.4,0.5,0.2\succ ),({\widehat{P}}_{2}/\prec 0.2,0.7,0.3\succ ),\\ \displaystyle ({\widehat{P}}_{3}/\prec 0.6,0.3,0.5\succ ),({\widehat{P}}_{4}/\prec 0.4,0.6,0.5\succ ) \end{array} \right\} \right) ,\\ \displaystyle \left( ({\zeta }_{3}/0.4,{\widehat{\Xi }}_{1},0),\Lambda ({\zeta }_{3}/0.4,{\widehat{\Xi }}_{1},0)= \left\{ \begin{array}{@{}l@{}} \displaystyle ({\widehat{P}}_{1}/\prec 0.7,0.8,0.3\succ ),({\widehat{P}}_{2}/\prec 0.9,0.2,0.6\succ ),\\ \displaystyle ({\widehat{P}}_{3}/\prec 0.3,0.7,0.6\succ ),({\widehat{P}}_{4}/\prec 0.8,0.1,0.2\succ ) \end{array} \right\} \right) ,\\ \displaystyle \left( ({\zeta }_{4}/0.5,{\widehat{\Xi }}_{2},1),\Lambda ({\zeta }_{4}/0.5,{\widehat{\Xi }}_{2},1)= \left\{ \begin{array}{@{}l@{}} \displaystyle ({\widehat{P}}_{1}/\prec 0.3,0.7,0.9\succ ),({\widehat{P}}_{2}/\prec 0.4,0.8,0.5\succ ),\\ \displaystyle ({\widehat{P}}_{3}/\prec 0.7,0.4,0.5\succ ),({\widehat{P}}_{4}/\prec 0.6,0.7,0.3\succ ) \end{array} \right\} \right) \end{array} \right\} . \end{eqnarray*}

In above set, we see that this element $({\widehat{P}}_{1}/\prec 0.2,0.3,0.4\succ )$ represents the collective information given by the decision makers having 0.2(20%) as a membership value, 0.3 (30%) as an ambiguous value and 0.4 (40%), a non-membership value to disease ${\widehat{P}}_{1}$ for the taking side of special’s judgement in this FpNHse-set, keeping in mind all subsequent estimates and their values are calculated in a manner similar to this.

## Proposed technique and algorithmic usage

**Figure 1:**
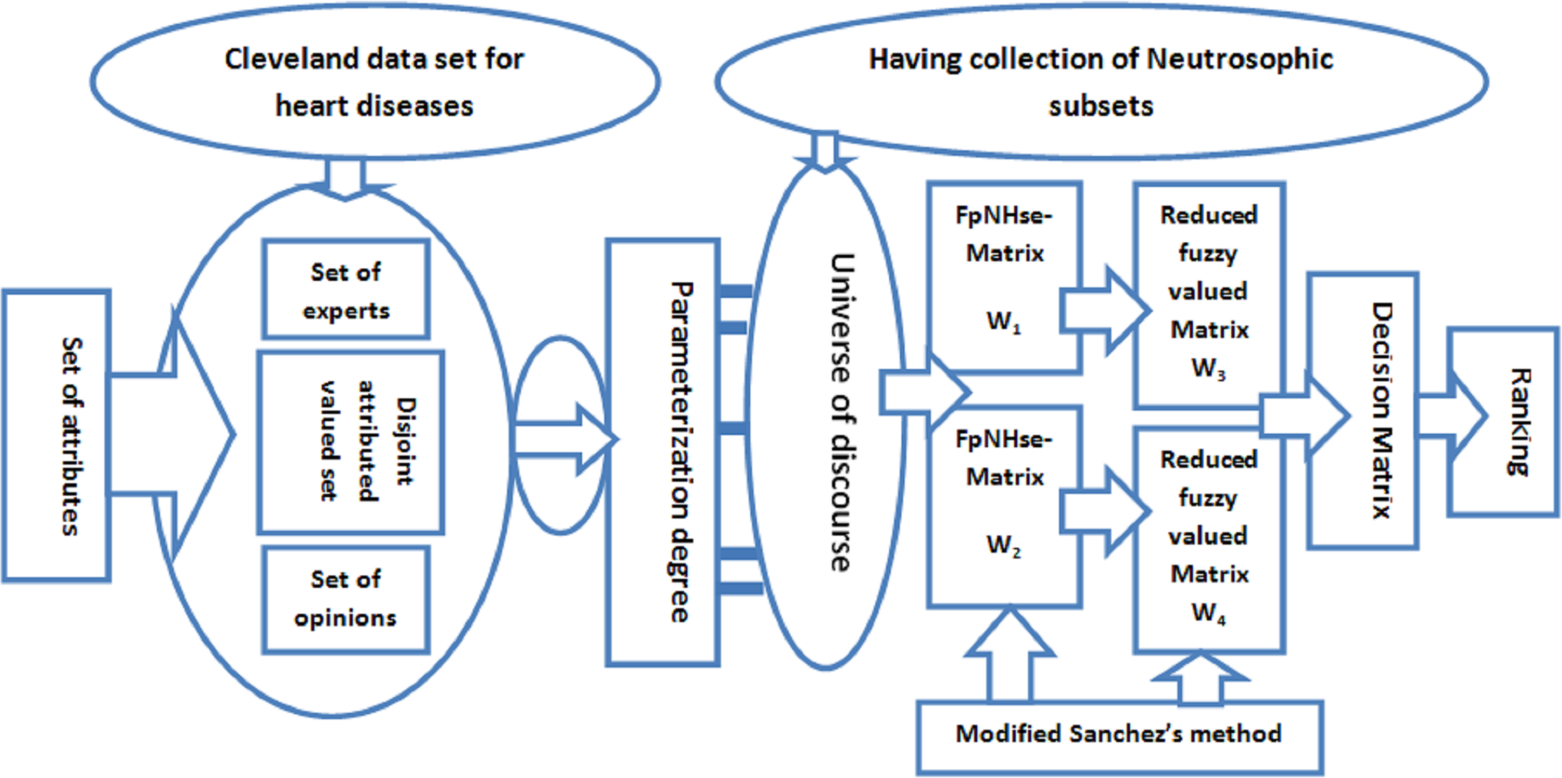
Explanation of methodology by pictorial representation.

The following study uses real data from the Cleveland data set (O’Brien et al., 2008) in the FpNHse-set environment to diagnose heart problems using the medical diagnosis approach of Sanchez (1979) with some minor adjustments. Figure 1 shows a visual depiction of the entire technique that was used to create the publication.

### Modified Sanchez’s method

A traditional way for connecting a model’s parameters, universal sets, and decision-makers’s perspectives is Sanchez’s method (Sanchez, 1979). To establish this relationship, it makes use of the idea of matrix theory. Although it is typically used for medicinal analysis, it may be used in any other situation with minor adjustments. It needs primary matrices, which are transitively built from characteristics, universal sets, and decision-makers’s viewpoints. We now provide this method with adjustments for our suggested model.

Let 𝔎 = 𝔨_1_ × 𝔨_2_ × 𝔨_3_ × ... × 𝔨_*q*_ = {*k*_1_, *k*_2_, *k*_3_...*k*_*q*_} be the store of pairs having attributed values, where 𝔨_*i*_ are non-overlapping attribute-valued collection with respect to the specified attributes of the Cleveland data set, and let 𝕄 = {*m*_1_, *m*_2_, *m*_3_, …, *m*_*p*_} be the initial universe containing a line of diseased persons. A panel of decision-makers $\Delta =\{ {\ddot{\Upsilon }}_{1},{\ddot{\Upsilon }}_{2},{\ddot{\Upsilon }}_{3}...{\ddot{\Upsilon }}_{r}\} $ is allowed to take part in the diagnosis. We have the corresponding matrix notations ${\mathcal{W}}_{1}=[{a}_{ij}]_{p&times; q}$ and ${\mathcal{W}}_{2}=[{b}_{ij}]_{q&times; r}$ for two FpNHse-sets. While ${\mathcal{W}}_{2}=[{b}_{ij}]_{p&times; q}$ is a matrix with FpNHse values allocated by specialists to the pairs of attributed values in 𝔎 as members, ${\mathcal{W}}_{1}=[{b}_{ij}]_{p&times; q}$ is a matrix with uncertain type grades matching to the pairs of attributed values in 𝔎 as elements. The matrices ${\mathcal{W}}_{1}=[{b}_{ij}]_{p&times; q}$ and ${\mathcal{W}}_{2}=[{b}_{ij}]_{q&times; r}$ are converted to ${\mathcal{W}}_{3}=[{b}_{ij}]_{p&times; q}$ and, ${\mathcal{W}}_{4}=[{b}_{ij}]_{q&times; r}$ respectively, once FpNHse values are reduced to fuzzy values. We may get the decision matrix ${\mathcal{W}}_{5}=[{b}_{ij}]_{q&times; r}$ by taking the (typical product of matrices) of 3rd and 4th matrices respectively.

### Cleveland data set

Heart issue diagnostic research is designed to use the Cleveland data set (UCI Machine Learning Repository, 2010), 303 diseased persons were investigated for the analysis of heart issues utilising 76 characteristics and five outcomes in this data collection (14 of which, however, can be used in experiments and analysis). In the Cleveland data set there are 14 significant parameters related to heart disease. Nine of the most pertinent attributes are taken into consideration while choosing six people for heart disease diagnosis, bearing for the further classification of properties into their corresponding disjoint set of attribute-valued. Only the 9 parameters from the Cleveland data set are selected from the total of 14 parameters. This is so that parameters can be further categorised into their relevant sub-parametric values in the form of disjoint sets, as required by the suggested structure FpNHse-set. Six diseased persons have been selected because Sanchez’s technique, and including extra diseased persons could have led to ant complicated problem. But, with the appropriate planning, such complexity might be overcome. These nine qualities are explained in Table 2 along with the suggested values for each (data set). Note:

**Table 2:** Parameters of the Cleveland data set and their values.

Parameter	Full name	Values of the parameters
thal	3 = normal; 6 = fixed defect; 7 = reversible defect	1. normal, 2. fixed defect, 3. reversible defect
trestpbs	Resting blood pressure (mm Hg)	90–200mm Hg
slope	The slope of the peak exercise ST segment	1. upsloping, 2. flat, 3. downsloping
age	Age in years	0–20, 21–40, 41–60, Above 60
cp	Chest pain type	1. Typical angina, 2. atypical angina, 3. non-anginal pain, 4. asymptomatic
chol	Serum cholesterol (mg/dL)	126–564 mg/dL
fbs	Fasting blood sugar	(120 mg/dL) 120 mg/dL
Oldpeak	ST depression induced by exercise relative to rest	0.0–5.6
Thalach	Maximum heart rate achieved	71–195

1. The Cleveland data set is one of the prominent large datasets. This consists of observations obtained by considering 303 patients with 76 evaluating indicators (attributes or characteristics). The necessary condition has already been discussed by many researchers based on this large dataset. These researches are not based on the concept of classification, that’s why the whole population has been included (all patients and attributes), *i.e.,* reduction in the size of the dataset is not the demand of such research.
2. However, the sufficient condition (conversely) is interpreted in such a way that the universal set of 303 patients has been categorized into six disjoint groups based on the evaluating indicators. Consequently, six patients (Patient 1, Patient 2, Patient 24, Patient 25, Patient 75, and Patient 303) are selected from these groups by taking one from each group. The same mechanism has been adopted while screening out the number of attributes and sub-attributes, *i.e.,* the selection of 9 attributes out of 76 attributes.
3. As the proposed approach is in fact a part of medical classification which itself demands statistical data based on sampling. That’s why the authors have reduced the size of the dataset from (*a* = 303; *b* = 76) to (*a* = 6; *b* = 9) to fulfill the demand of the medical classification scenario. In the mentioned pair, “a” stands for the number of patients considered for diagnosis, and “b” stands for the number of attributes considered for such evaluation.

### Operational role of selected attributes

To support their choice for the diagnosis of cardiac disorders, the operational role of the chosen traits is addressed in this section of the paragraph:

1. **Age**: Heart illnesses are independently at risk due to ageing. Although elderly adults (60 years or more) have a higher chance of developing heart disease, younger people may also be at risk if certain additional variables are present. Four categories of ageing have been established by medical professionals: up to 20, 40, 60 and above.
2. **Chest pain type**: It may be the utmost common logic for people to go to the trauma department is chest pain. Depending on the person, it varies. It also varies in terms of quality, force, span, and area. It could be a mild pain or an acute, painful feeling. It can be a sign of an actual cardiac condition. It could have also been induced by a variety of harmless typical factors. Atypical angina (ATA), non-anginal pain, typical angina (TA), and asymptomatic chest pain are different types of heart-related chest pain (AM). Angina is typically characterised by substernal chest pain or discomfort that is (1) brought on by physical activity or mental stress and (2) alleviated by rest or nitroglycerin (or both). When two of the three traditional angina criteria are met, atypical (probable) angina chest pain is present. Non-anginal pain is used to identify hospitalised individuals or showing signs of a myocardial ischemia.
3. **Resting blood pressure**: The pressure that the blood applies to the arterial walls is known as blood pressure. Systolic and diastolic pressure are additional categories for such pressure. The first occurs when the heart releases blood into the blood vessels, while the second occurs when the heart is resting and occurs inside the arteries. When the blood pressure is abnormally high during cardiac compression or when the arteries are relaxing, this condition is known as hypertension. There could be more resistance to blood flow in the arteries. In mm Hg, both pressures are expressed. Systolic blood pressure should be less than 120 and diastolic should be less than 80 (120/80). If your blood pressure is higher than 120 to 129, and diastolic below 80.
4. **Serum cholesterol**: A type of fat is cholesterol. A lipid is another name for it. It circulates in our bloodstream as tiny particles encased in proteins. Lipoproteins are the name given to these bundles. One of the main types of lipoproteins in human blood is LDL. High-density lipoproteins (HDLs) are the other main type. Triglycerides, a third class of lipid, also circulate in our blood. Our complete blood cholesterol, also known as serum cholesterol, is calculated by estimating our HDL (the “good” cholesterol), LDL (the “bad” cholesterol), and triglycerides. Although our bodies require cholesterol to create healthy cells, having too much of it can increase our chance of developing heart disease. Our blood arteries may become fatty with excessive cholesterol levels. Over time, these deposits thicken and restrict the amount of blood that can pass through our arteries. HDL and LDL cholesterol levels are added, along with 20 percent of triglycerides, to determine serum cholesterol levels. It fluctuates between 126 and 564 mg/dL.
5. **Fasting blood sugar** Due to the “stress reaction”, a significant portion of persons with heart disease have elevated glucose levels. This implies that those who do not have diabetes may nevertheless have high blood sugar. A healthy person’s range is between 120 mg/dL to 140 mg/dL.
6. **Maximum heart rate achieved**: In individuals with ischemic heart disease, heart rate plays a significant role in determining oxygen consumption. Its highest value, which can be reached, falls between 71 and 195 b/m.
7. **Old peak and slope**: Exercise-induced Shock, Toxicity depression compared to rest is regarded as a trustworthy electrocardiogram (ECG) finding for the identification of obstructive coronary diseases. It ranges from 0.0 to 0.5 mm and is measured in mm. The exercise’s peak’s incline The three types of ST-segments are up sloping, flat (horizontal), and down sloping.
8. **Thal**: This is brought on by the blood disorder thalassemia. Four categories can be made from it: null, fixed defect, no blood flow in some heart locations, normal blood flow, and reversible defect, blood flow is present but is aberrant. It’s common practise to ignore the first group when diagnosing heart issues.

### Parameterized grades corresponding to selected attributes

The mandated values (values assigned in the Cleveland data set) of the selected characteristics are converted into the associated parameterized grades in this stage using an appropriate transformational criterion. The potential grade for each attribute is determined by dividing its prescribed value by its highest prescribed value. The maximum prescribed value for chest pain is 4 and the other attributes in the Cleveland data set have highest values. After this the fuzzy parameterized valued is obtained by subtracting the highest value from the lowest value and divide by 2. This is shown in Table 3. It can viewed by the following formula.

Let ${\ddot{\bigvee }}^{i}$ be the multi-argument multi-decisive tuples corresponding non-overlapping parametric sub classes and parameters then their corresponding approximated fuzzy grades are denoted by $\mu ({\ddot{\bigvee }}^{i})$. Let ${\mu }_{max}(\ddot{\bigvee })$ and ${\mu }_{min}(\ddot{\bigvee })$ be the maximum and minimum approximated fuzzy grades then fuzzy parameterized grade corresponding to $\ddot{\bigvee }$ can be computed with the help of the following formulation

(1) \begin{eqnarray*}\chi (\ddot{\bigvee })= \frac{{\mu }_{max}(\ddot{\bigvee })-{\mu }_{min}(\ddot{\bigvee })}{2} .\end{eqnarray*}

### Scenario and statement of the problem

The clinical diagnosis of particular diseases through mathematical modelling is a topic that scholars are getting more and more interested in. This modelling may make use of real or made-up facts or information. Since the creation of N-set, researchers have become interested in neutrosophic modelling for clinical analysis with ambiguous circumstances. Numerous modifications and generalisations have been made to N-sets. The FpNHse-set is one of them. It not only generalises the existing structures but also addresses their flaws in terms of how they handle the use of parameterized grades collectively and the new classification of characteristics into disjoint sets of attributed values. There are a few research that have been published in the study that parameterize numerical structures for clinical analysis using real data and fuzzy set extensions. This study used fresh conditions of the FpNHse-set for the medical analysis of certain people with cardiac problems using actual data from the Cleveland data set.

**Table 3:** Transformed values from ordinary to fuzzy values.

Name of parameter	Given value in data set	Transferred value
Chest pain type	1,2,3,4	0.25,0.50,0.75,1
Resting blood pressure	90–200	0.45–1
slope	1,2,3	0.33, 0.66.1
age	0–20, 21–40, 41–60,	0–0.33, 0.35–0.67, 0.68–1.00
Thal	3,6,7	0.4286, 0.8571, 1
Serum cholesterol	126–564	0.2234–1
Fasting blood sugar	0, 120	0,1
Old peak	0.0-5.6	0.0, 1
Thalach	71-195	0.36–1

### Proposed algorithm and implementation

**Figure 2:**
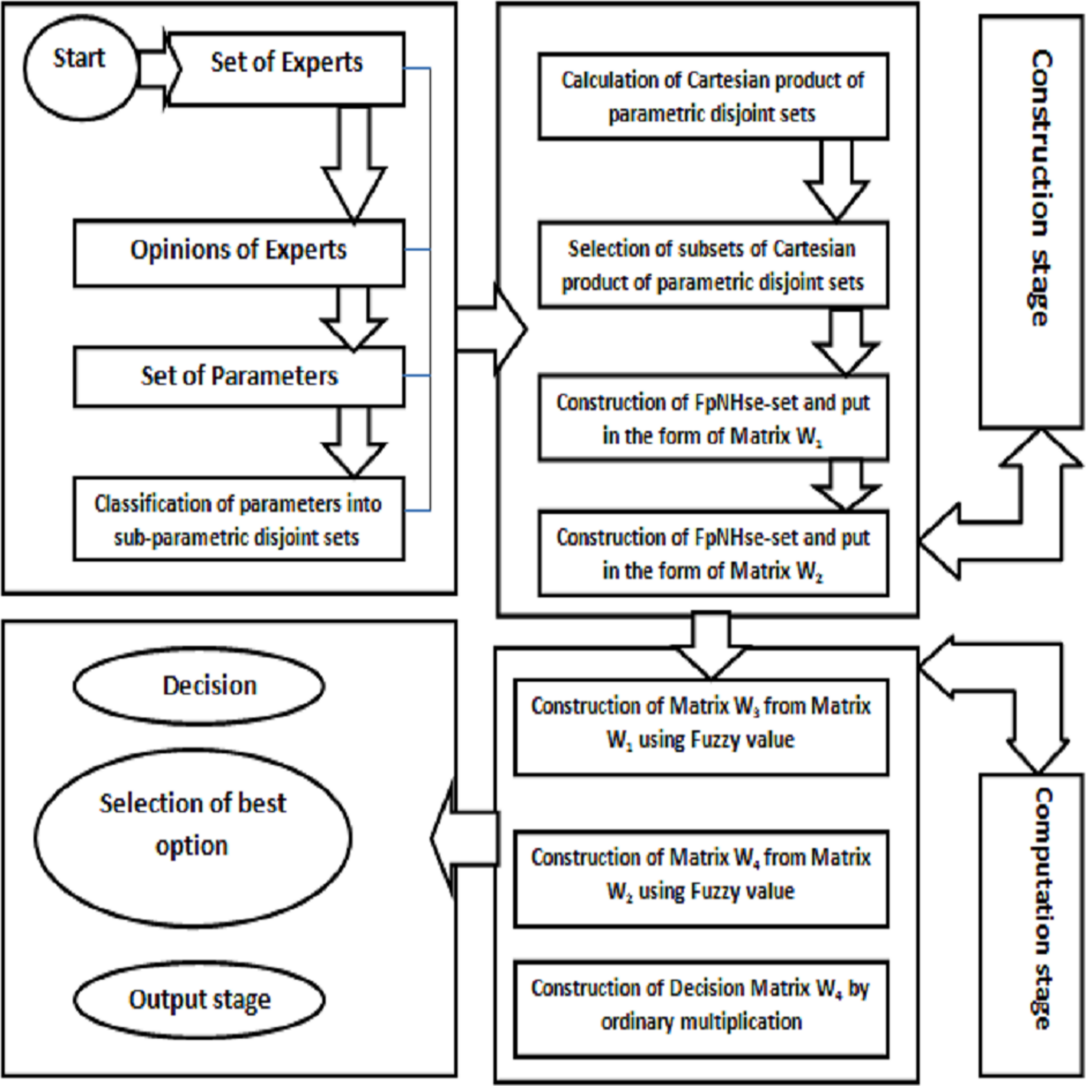
Flowchart of algorithm.

**Figure 3:**
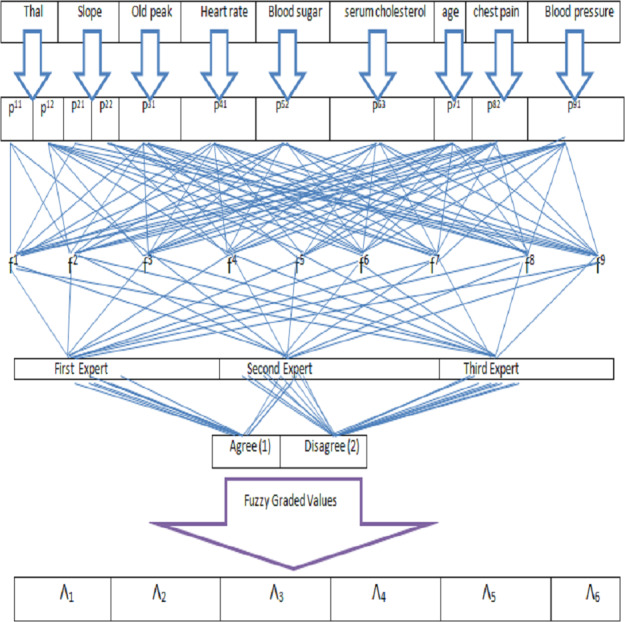
Hierarchical structure of decision problem.

The idea of aggregating the FpNHse-set is used in this part to suggest an algorithm for the diagnosis of heart disorders in patients. Different types of software and programming languages are used in multidisciplinary research, for example, mathematicians prefer LATEX, MATLAB, MSWORD, etc., computer science researchers prefer Python, C++, etc, for computer researchers, while scientific workspace for physicists and chemists. Online, there are numerous converters available for converting these programmes and codes amongst one other. It is important to use an approach that is simple to grasp but thorough to describe the suggested algorithm’s procedural flow so that researchers can adapt it to their area of interest and improve the comprehension of the suggested work. Due to the multidisciplinary nature of the proposed study, a straightforward explanation of the proposed algorithm’s procedural flow was established for the benefit of the researchers (readers). The aforementioned technique is demonstrated using the example below. Figure 2 shows a flowchart that summarises the processes of Algorithm 1 and Fig. 3 presents the hierarchical structure of the proposed decision design.


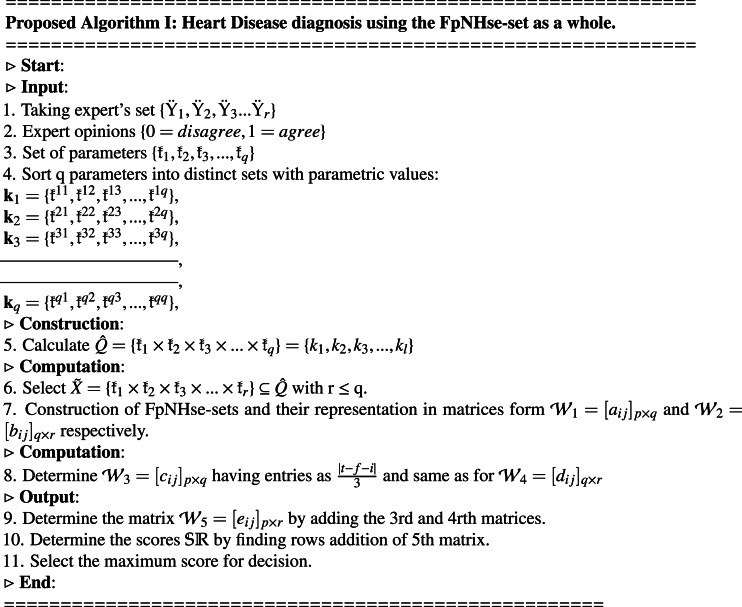

Example 4.1 By using the data from Cleveland, select six patients for heart issue analysis; these will make up the set $\mathbf{U}=\{ {\hat {\Lambda }}_{1},{\hat {\Lambda }}_{2},{\hat {\Lambda }}_{24},{\hat {\Lambda }}_{25},{\hat {\Lambda }}_{75},{\hat {\Lambda }}_{303}\} $. Let $\mathbf{D}=\{ {\hat {\Phi }}_{1},{\hat {\Phi }}_{2},{\hat {\Phi }}_{3},{\hat {\Phi }}_{4}\} $ represents the group of heart specialist (experts) who will evaluate the diagnosing course and their decisions ⨆ = {0 = *disagree*, 1 = *agree*} and the set of parameters is $\mathbf{L}=&lcub; {\mathcal{L}}^{1},{\mathcal{L}}^{2},{\mathcal{L}}^{3},{\mathcal{L}}^{4},{\mathcal{L}}^{5},{\mathcal{L}}^{6},{\mathcal{L}}^{7},{\mathcal{L}}^{8},{\mathcal{L}}^{9}&rcub; $ where each parameters has its own identity such as ${\mathcal{L}}^{1}$ = thal, ${\mathcal{L}}^{2}$ =slope, ${\mathcal{L}}^{3}$ = old peak, ${\mathcal{L}}^{4}$ = maxi. hear rate gained, ${\mathcal{L}}^{5}$ = fasting blood sugar, ${\mathcal{L}}^{6}$ = serum cholesterol, ${\mathcal{L}}^{7}$ = age, ${\mathcal{L}}^{8}$ = chest pain, ${\mathcal{L}}^{9}$ = blood pressure. The disjoint sets having attribute-valued corresponding to used attributes are: ${\mathcal{L}}^{1}$ = {*p*^11^ = *reversibledefect*, *p*^12^ = *normal*}, ${\mathcal{L}}^{2}$ = $&lcub; {p}^{21}=downsloping,{p}^{22}=upsloping,{p}^{23}=flate&rcub; ,{\mathcal{P}}^{3}$ = $&lcub; {p}^{31}=1.2,{p}^{32}=3.7&rcub; ,{\mathcal{P}}^{4}$ = $&lcub; {p}^{41}=81,{p}^{42}=141&rcub; ,{\mathcal{P}}^{5}$ = $&lcub; {p}^{51}=120~\mathrm{mg/dL}&rcub; ,{\mathcal{L}}^{6}$ = {*p*^61^ = 210 mg/dl, *p*^62^ = 320 mg/dl, *p*^63^ = 320 mg/dl}, ${\mathcal{L}}^{7}$ = {*p*^71^ = 1*stcategory*, *p*^72^ = 2*ndcategory*, *p*^73^ = 3*rdcatgory*, *p*^74^ = 4*r*^*th*^*catgory*},${\mathcal{L}}^{8}$ = {*p*^81^ = *typicalangina*, *p*^82^ = 2*ndatypicalangina*, *p*^83^ = *non* − *anginalpain*, *p*^84^ = *asymptomatic*}, ${\mathcal{L}}^{9}$ = {*p*^91^ = 110 mmHg, *p*^92^ = 150 mmHg, *p*^93^ = 180 mmHg}.

**Construction stage**:

Now, **T** = ${\mathcal{L}}^{1}&times; {\mathcal{L}}^{2}&times; {\mathcal{L}}^{3}&times; {\mathcal{L}}^{4}&times; {\mathcal{L}}^{5}&times; {\mathcal{L}}^{6}&times; {\mathcal{L}}^{7}&times; {\mathcal{L}}^{8}&times; {\mathcal{L}}^{9}=2&times; 3&times; 2&times; 2&times; 1&times; 3&times; 4&times; 4&times; 3=3456$and this will be 9-tuple element of **T**. After having a strong discussion with heart specialists, certain parameters have been selected for further proceedings such as *p*^11^ and *p*^12^ in ${\mathcal{L}}^{1}$ and similarly *p*^21^ and *p*^22^ in ${\mathcal{L}}^{2}$, *p*^31^ in ${\mathcal{L}}^{3},{p}^{41}$ in ${\mathcal{L}}^{4},{p}^{52}$ in ${\mathcal{L}}^{5},{p}^{63}$ in ${\mathcal{L}}^{6},{p}^{71}$ in ${\mathcal{L}}^{7},{p}^{82}$ in ${\mathcal{L}}^{8},{p}^{91}$ in ${\mathcal{L}}^{9}$. We take $\mathbf{F}=\{ {\ddot{\infty }}_{1},{\ddot{\infty }}_{2},{\ddot{\infty }}_{3},{\ddot{\infty }}_{4},{\ddot{\infty }}_{5},{\ddot{\infty }}_{6},{\ddot{\infty }}_{7},{\ddot{\infty }}_{8}\} $ as subset of **T** in which every element has 9-tuple element. Now $\ddot{\Delta }=\mathbf{F}\times \mathbf{D}\times \mathbf{N}$. After these Cartesian product of experts set with their opinions and **F**, we get 64 pairs and each pair is a triplet. Let $\Xi =\{ {\ddot{\bigvee }}^{1},{\ddot{\bigvee }}^{2},{\ddot{\bigvee }}^{3},{\ddot{\bigvee }}^{4},{\ddot{\bigvee }}^{5},{\ddot{\bigvee }}^{6},{\ddot{\bigvee }}^{7},{\ddot{\bigvee }}^{8}\} $ be the subset of $\ddot{\Delta }$.

**Next Stage**:

In this stage two FpNHse-sets are developed in the form of matrices. The Tables 4 and 5, respectively are selected to show these matrices. Table 6 presents the mechanism for the determination of fuzzy parameterized valued for multi argument tuples.

**Next stage step 7 and 8**:

The new matrices ${\mathcal{W}}_{3}$ and ${\mathcal{W}}_{4}$ so acquired are provided in Tables 7 and 8, respectively. Since the number used in ${\mathcal{W}}_{1}$ and ${\mathcal{W}}_{2}$ are actually the values of FpNHse-set, these important values are made to change in fuzzy values with the help of method $ \frac{ \left\vert t-f-i \right\vert }{3} $. To ensure a discrete judgement, FpNHse values are transformed to fuzzy values. Fuzzy parameterized values for the sub-parametric tuples have been calculated by subtracting the highest fuzzy value from the lowest fuzzy value and divided by 2. Then these values have been used in Tables 7 and 8 respectively to form new matrices ${\mathcal{W}}_{4}$ and ${\mathcal{W}}_{5}$. After this, these values are multiplied with column of ${\mathcal{W}}_{4}$ to form a new matrix ${\mathcal{W}}_{6}$ and row wise multiplied in matrix ${\mathcal{W}}_{5}$ to form a matrix ${\mathcal{W}}_{7}$. These two matrices have been shown in Tables 9 and 10.

**Table 4:** Matrix notation of FpNHse-set.

${\mathcal{W}}_{1}$	${\ddot{\bigvee }}^{1}$	${\ddot{\bigvee }}^{2}$	${\ddot{\bigvee }}^{3}$	${\ddot{\bigvee }}^{4}$
${\hat {\Lambda }}_{1}$	〈0.4, 0.6, 0.1〉	〈0.3, 0.4, 0.5〉	〈0.4, 0.6, 0.1〉	〈0.6, 0.2, 0.3〉
${\hat {\Lambda }}_{2}$	〈0.4, 0.5, 0.3〉	〈0.6, 0.1, 0.9〉	〈0.8, 0.4, 0.2〉	〈0.5, 0.4, 0.2〉
${\hat {\Lambda }}_{24}$	〈0.3, 0.7, 0.1〉	〈0.2, 0.7, 0.3〉	〈0.5, 0.3, 0.4〉	〈0.9, 0.3, 0.1〉
${\hat {\Lambda }}_{25}$	〈0.4, 0.6, 0.1〉	〈0.1, 0.3, 0.9〉	〈0.8, 0.1, 0.4〉	〈0.2, 0.7, 0.3〉
${\hat {\Lambda }}_{75}$	〈0.3, 0.9, 0.8〉	〈0.2, 0.5, 0.6〉	〈0.9, 0.3, 0.4〉	〈0.8, 0.3, 0.1〉
${\hat {\Lambda }}_{303}$	〈0.2, 0.5, 0.6〉	〈0.3, 0.4, 0.7〉	〈0.5, 0.4, 0.2〉	〈0.3, 0.3, 0.5〉

${\mathcal{W}}_{1}$	${\ddot{\bigvee }}^{5}$	${\ddot{\bigvee }}^{6}$	${\ddot{\bigvee }}^{7}$	${\ddot{\bigvee }}^{8}$
${\hat {\Lambda }}_{1}$	〈0.7, 0.3, 0.1〉	〈0.4, 0.2, 0.5〉	〈0.4, 0.6, 0.1〉	〈0.6, 0.2, 0.3〉
${\hat {\Lambda }}_{2}$	〈0.4, 0.5, 0.3〉	〈0.6, 0.1, 0.9〉	〈0.8, 0.4, 0.2〉	〈0.5, 0.4, 0.6〉
${\hat {\Lambda }}_{24}$	〈0.9, 0.4, 0.2〉	〈0.4, 0.3, 0.5〉	〈0.7, 0.3, 0.8〉	〈0.3, 0.4, 0.4〉
${\hat {\Lambda }}_{25}$	〈0.6, 0.3, 0.2〉	〈0.7, 0.6, 0.5〉	〈0.6, 0.8, 0.2〉	〈0.4, 0.6, 0.8〉
${\hat {\Lambda }}_{75}$	〈0.5, 0.5, 0.1〉	〈0.9, 0.3, 0.8〉	〈0.9, 0.6, 0.5〉	〈0.6, 0.7, 0.1〉
${\hat {\Lambda }}_{303}$	〈0.1, 0.3, 0.9〉	〈0.2, 0.4, 0.5〉	〈0.6, 0.7, 0.6〉	〈0.5, 0.3, 0.6〉

**Table 5:** Matrix notation FpNHse-set.

${\mathcal{W}}_{2}$	*DM*_1_	*DM*_2_	*DM*_3_	*DM*_4_
${\ddot{\bigvee }}^{1}$	〈0.6, 0.3, 0.2〉	〈0.4, 0.2, 0.5〉	〈0.4, 0.3, 0.5〉	〈0.6, 0.2, 0.3〉
${\ddot{\bigvee }}^{2}$	〈0.4, 0.5, 0.3〉	〈0.6, 0.6, 0.9〉	〈0.9, 0.4, 0.2〉	〈0.5, 0.4, 0.2〉
${\ddot{\bigvee }}^{3}$	〈0.5, 0.6, 0.1〉	〈0.7, 0.3, 0.1〉	〈0.5, 0.3, 0.3〉	〈0.7, 0.3, 0.1〉
${\ddot{\bigvee }}^{4}$	〈0.9, 0.3, 0.1〉	〈0.5, 0.8, 0.4〉	〈0.2, 0.7, 0.5〉	〈0.4, 0.5, 0.3〉
${\ddot{\bigvee }}^{5}$	〈0.2, 0.3, 0.8〉	〈0.9, 0.3, 0.1〉	〈0.2, 0.9, 0.1〉	〈0.6, 0.3, 0.1〉
${\ddot{\bigvee }}^{6}$	〈0.9, 0.2, 0.1〉	〈0.2, 0.7, 0.6〉	〈0.4, 0.3, 0.6〉	〈0.2, 0.9, 0.2〉
${\ddot{\bigvee }}^{7}$	〈0.7, 0.3, 0.5〉	〈0.3, 0.6, 0.2〉	〈0.7, 0.4, 0.1〉	〈0.3, 0.7, 0.1〉
${\ddot{\bigvee }}^{8}$	〈0.4, 0.5, 0.3〉	〈0.7, 0.3, 0.5〉	〈0.2, 0.3, 0.6〉	〈0.4, 0.3, 0.4〉

**Table 6:** Criteria for fuzzy parameterized value for sub-parametric tuples.

Sub-parameter tuples	Given value in data set	Transformed value	Fuzzy parameterized value
${\ddot{\bigvee }}^{1}$	1,2,3,4	0.25,0.50,0.75,1	$\chi ({\ddot{\bigvee }}^{1})$= 1-0.25/2=0.38
${\ddot{\bigvee }}^{2}$	90–200	0.45–1	$\chi ({\ddot{\bigvee }}^{2})$= 1-0.45/2=0.28
${\ddot{\bigvee }}^{3}$	1,2,3	0.33, 0.66.1	$\chi ({\ddot{\bigvee }}^{2})$= 1-0.33/2=0.33
${\ddot{\bigvee }}^{4}$	71-195	0.36–1	$\chi ({\ddot{\bigvee }}^{2})$= 1-0.36/2=0.32
${\ddot{\bigvee }}^{5}$	3,6,7	0.43, 0.86, 1	$\chi ({\ddot{\bigvee }}^{2})$= 1-0.43/2=0.29
${\ddot{\bigvee }}^{6}$	126–564	0.22–1	$\chi ({\ddot{\bigvee }}^{2})$= 1-0.22/2=0.36
${\ddot{\bigvee }}^{7}$	0, 120	0,1	$\chi ({\ddot{\bigvee }}^{2})$= 1-0/2=0.5
${\ddot{\bigvee }}^{8}$	0.0-5.6	0.0, 1	$\chi ({\ddot{\bigvee }}^{2})$= 1-0/2=0.5

**Step 9**:

The matrix ${\mathcal{W}}_{8}$ of rank 6 × 4 is created by multiplying ${\mathcal{W}}_{6}$ and ${\mathcal{W}}_{7}$ using the traditional concept of matrix multiplication. This matrix is shown in Table 11.

**Figure 4:**
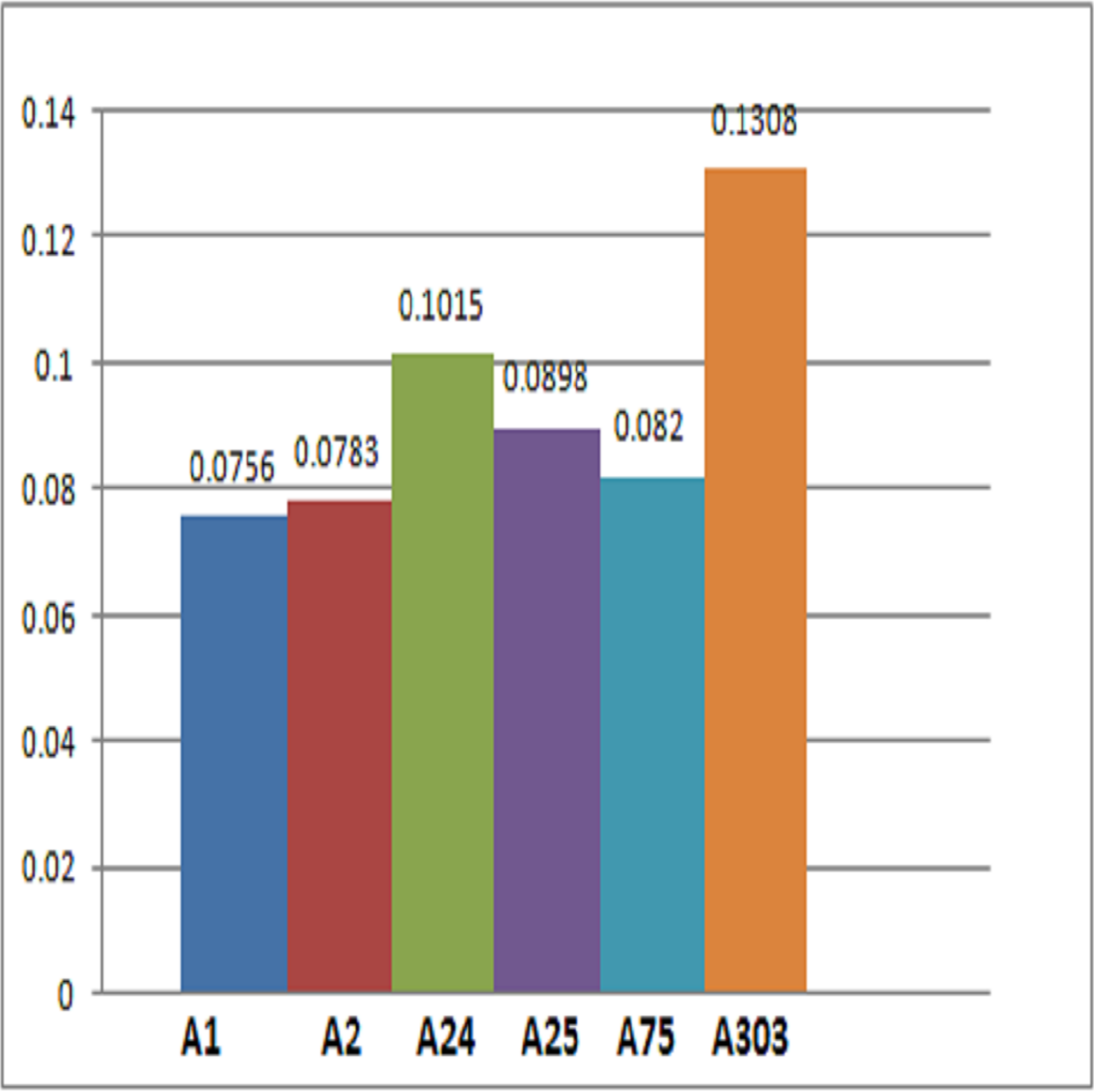
Representation of alternatives.

**Final stage step 10**:

Final scores are obtained by adding the each row. Figure 4 and Table 12 demonstrate that patient ${\hat {\Lambda }}_{303}$ is more likely than the rest to have cardiac disease.

**Table 7:** Matrix notation of FpNHse-set.

${\mathcal{W}}_{4}$	${\ddot{\bigvee }}^{1}/0.38$	${\ddot{\bigvee }}^{2}/0.28$	${\ddot{\bigvee }}^{3}/0.33$	${\ddot{\bigvee }}^{4}/0.32$	${\ddot{\bigvee }}^{5}/0.29$	${\ddot{\bigvee }}^{6}/0.36$	${\ddot{\bigvee }}^{7}/0.5$	${\ddot{\bigvee }}^{8}/0.5$
${\hat {\Lambda }}_{1}$	0.1	0.2	0.1	0.03	0.1	0.1	0.1	0.03
${\hat {\Lambda }}_{2}$	0.13	0.13	0.07	0.03	0.13	0.13	0.07	0.17
${\hat {\Lambda }}_{24}$	0.17	0.27	0.07	0.17	0.1	0.13	0.13	0.17
${\hat {\Lambda }}_{25}$	0.17	0.37	0.1	0.27	0.03	0.13	0.13	0.03
${\hat {\Lambda }}_{75}$	0.47	0.3	0.07	0.13	0.03	0.07	0.07	0.07
${\hat {\Lambda }}_{303}$	0.3	0.27	0.03	0.17	0.37	0.23	0.23	0.13

**Table 8:** Matrix notation FpNHse-set.

${\mathcal{W}}_{5}$	*DM*_1_	*DM*_2_	*DM*_3_	*DM*_4_
${\ddot{\bigvee }}^{1}/0.38$	0.03	0.1	0.2	0.03
${\ddot{\bigvee }}^{2}/0.28$	0.13	0.3	0.23	0.03
${\ddot{\bigvee }}^{3}/0.33$	0.07	0.3	0.03	0.1
${\ddot{\bigvee }}^{4}/0.32$	0.17	0.23	0.03	0.13
${\ddot{\bigvee }}^{5}/0.29$	0.3	0.17	0.27	0.07
${\ddot{\bigvee }}^{6}/0.36$	0.2	0.37	0.17	0.3
${\ddot{\bigvee }}^{7}/0.5$	0.03	0.17	0.13	0.17
${\ddot{\bigvee }}^{8}/0.5$	0.13	0.03	0.23	0.17

**Table 9:** Matrix notation of FpNHse-set.

${\mathcal{W}}_{6}$	${\ddot{\bigvee }}^{1}$	${\ddot{\bigvee }}^{2}$	${\ddot{\bigvee }}^{3}$	${\ddot{\bigvee }}^{4}$	${\ddot{\bigvee }}^{5}$	${\ddot{\bigvee }}^{6}$	${\ddot{\bigvee }}^{7}$	${\ddot{\bigvee }}^{8}$
${\hat {\Lambda }}_{1}$	0.038	0.056	0.033	0.0096	0.029	0.036	0.05	0.015
${\hat {\Lambda }}_{2}$	0.0494	0.0364	0.0231	0.0096	0.0377	0.0468	0.035	0.085
${\hat {\Lambda }}_{24}$	0.0646	0.0756	0.0231	0.0544	0.029	0.0468	0.065	0.085
${\hat {\Lambda }}_{25}$	0.0646	0.1036	0.033	0.0864	0.0087	0.0468	0.065	0.015
${\hat {\Lambda }}_{75}$	0.1786	0.084	0.0231	0.0416	0.0087	0.0252	0.035	0.035
${\hat {\Lambda }}_{303}$	0.0114	0.0756	0.0099	0.0544	0.1073	0.0828	0.115	0.065

**Table 10:** Matrix notation FpNHse-set.

${\mathcal{W}}_{7}$	*DM*_1_	*DM*_2_	*DM*_3_	*DM*_4_
${\ddot{\bigvee }}^{1}$	0.0114	0.038	0.076	0.0114
${\ddot{\bigvee }}^{2}$	0.0364	0.084	0.0644	0.0084
${\ddot{\bigvee }}^{3}$	0.0231	0.099	0.0099	0.033
${\ddot{\bigvee }}^{4}$	0.0544	0.0746	0.0096	0.0416
${\ddot{\bigvee }}^{5}$	0.087	0.0493	0.0783	0.0203
${\ddot{\bigvee }}^{6}$	0.072	0.1332	0.0612	0.108
${\ddot{\bigvee }}^{7}$	0.015	0.085	0.065	0.085
${\ddot{\bigvee }}^{8}$	0.065	0.015	0.115	0.085

**Table 11:** Matrix representation of ${\mathcal{W}}_{5}={\mathcal{W}}_{3}&times; {\mathcal{W}}_{4}$.

${\mathcal{W}}_{8}$	*DM*_1_	*DM*_2_	*DM*_3_	*DM*_4_
${\hat {\Lambda }}_{1}$	0.0260	0.0208	0.0164	0.0124
${\hat {\Lambda }}_{2}$	0.0156	0.0203	0.0243	0.0181
${\hat {\Lambda }}_{24}$	0.0194	0.0296	0.0297	0.0228
${\hat {\Lambda }}_{25}$	0.0160	0.0333	0.0222	0.0183
${\hat {\Lambda }}_{75}$	0.0133	0.0265	0.0281	0.0141
${\hat {\Lambda }}_{303}$	0.0273	0.0389	0.0348	0.0298

## Discussion, and sensitivity analysis and comparison analysis

Numerous researchers have already studied the medical diagnosis of various diseases using models that resemble neutrosophic sets in the literature. These methods employ hypothetical information with broad conclusions. Instead, the suggested study used the Cleveland data set, a genuine data collection, to identify and assess the risk of cardiac illnesses. In fact, present techniques overlook the scenario of medical diagnosis, which necessitates more classification of certain characters into their relevant values of sub-parameters and these values are found in sets with no common elements. Such a classification provides accurate outcomes and judgements. Meanwhile, Sanchez’s approach, which is simple but effective, was used for this diagnosis to reduce the computational complexity. As previously mentioned, the Cleveland data set has 74 attributes, and it would be difficult to take them all into account for the computations. As a result, only the nine attributes that played the most important roles were selected, after consulting with medical experts. According to the chosen data set, the values of sub-parameters and nature of these qualities are real. Following this debate, the primary benefits of the suggested study are listed as follows:

**Table 12:** Final score of the alternatives.

Alternatives	Final score
${\hat {\Lambda }}_{1}$	0.0260 + 0.0208 + 0.0164 + 0.0124 = 0.0756
${\hat {\Lambda }}_{2}$	0.0156 + 0.0203 + 0.0243 + 0.0181 = 0.0783
${\hat {\Lambda }}_{24}$	0.0194 + 0.0296 + 0.0297 + 0.0228 = 0.1015
${\hat {\Lambda }}_{25}$	0.0160 + 0.0333 + 0.0222 + 0.0183 = 0.0898
${\hat {\Lambda }}_{75}$	0.0133 + 0.0265 + 0.0281 + 0.0141 = 0.0820
${\hat {\Lambda }}_{303}$	0.0273 + 0.0389 + 0.0348 + 0.0298 = 0.1308

1. The adopted method used the FpNHse-set and parameterization to address the issues with modern decision-making. The alleged parameterized degree mimics the ambiguous nature of the level of acknowledgment; as a result, this has extraordinary potential for legitimacy inside the computational domain.
2. The Cleveland data set’s attributes and their sub-attributive values with real values were converted to fuzzy memberships using the proper transformation criteria.
3. The emphasis on thorough observation of the parameters and their corresponding sub-parametric values in the proposed model offers better, more dependable, and adaptable findings from medical doctors as decision-makers.

Because no researcher has previously used the suggested model, the FpNHse-set, to identify cardiac issues, the statistical outcomes of the recent work are not comparable with any previous structure. Though, some characteristics, including the indeterminacy score (IS), membership score (MS), non-membership score (NMS), score of parameterized (SOP), single argument-function (SIAF), multi-argument-function (MIAF), and expert’s opinions (EOP) are thought to be sufficient for comparing the proposed model with the most pertinent keep going structures. It is common to see in multi-attribute decision-making that some experts choose to avoid making any expert judgements on the characteristics for the things beneath study. This impartiality of the decision-makers is ensured by the incorporation of the IS. Similar to this, the SOP assures that the level of acceptability of the received expert judgements. The absence of these characteristics could result in a biased judgement. Table 13 is chosen for the comparison of this structure, which demonstrates clearly that the suggested model meets all of the requirements individually in one model whereas the existing models are missing one or more features. Therefore, it is safe to say that the proposed model is more generalised, adaptable, and trustworthy than the ones that now exist. The symbols ⇑ and ⇓ in Table 13 stand for Yes and No, respectively.

**Table 13:** Comparison analysis of FpNHse-set.

Author’s name	Structure	I.S	M.S	N.M.S	S.O.P	S.I.A.F	M.I.A.F	EOP
Debnath (2021)	FHs-set	⇓	⇑	⇑	⇓	⇑	⇑	⇓
Yolcu, Smarandache & Öztürk (2021)	IFHs-set	⇓	⇑	⇓	⇓	⇑	⇑	⇓
Cagman & Enginoglu (2011)	FP-FSS	⇓	⇑	⇑	⇑	⇑	⇓	⇓
Bashir & Salleh (2012)	FP-SES	⇓	⇑	⇓	⇑	⇑	⇓	⇓
Hazaymeh et al. (2012)	FP-FSES	⇑	⇑	⇓	⇑	⇑	⇓	⇓
Selvachandran & Salleh (2016)	FP-IFSES	⇓	⇑	⇓	⇑	⇑	⇓	⇓
Rahman et al. (2022a)	FPNHs-set	⇑	⇑	⇓	⇑	⇑	⇑	⇓
Proposed structure	FpNHse-set	⇑	⇑	⇓	⇑	⇑	⇑	⇑

In the Table 13, the arrows are used to indicate whether certain attributes or factors are present or not in the respective structures being compared. An upward arrow signifies the presence or existence of a particular attribute, while a downward arrow indicates the absence or non-existence of that attribute. In relation to the Table 13, the following is an explanation of the arrow symbols:

1. ⇑ (upward arrow): Represents the existence or favourable existence of a property. An upward arrow, for instance, indicates that a property has that property or demonstrates that property in the relevant structure or procedure.
2. ⇓ (downward arrow): Indicates a quality’s lack or unfavourable presence. If a property is shown by a downward arrow, it signifies that the relevant structure or method lacks the property or does not display the property.

The arrows represent the presence or absence of the matching attribute in the framework suggested by each author. The upward arrows indicate the presence of the attribute, while the downward arrows indicate its lack. The structure “FHs-set” in the row for “Debnath (2021)”, for instance, has an upward arrow next to “structure”, suggesting that it has the attribute of structure. In a similar manner, it has upward arrows for IS, MS, SIAF, and MIAF, signifying the existence of these traits, and downward arrows for NMS and EOP, signifying the absence of them. Anyone can determine if each attribute is present or absent in the comparing structures or techniques by interpreting the arrows in this way. The suggested structure is compared with the others structures based on ranking and is shown in Table 14. Some parameters like number of attributes (*NA*), number of patients (*NP*), Sanchez’s method, ranking, and use of sub-attributes (*UOSA*) are used for this comparison. Kirisci, Demir & Simsek (2021) and Kirisci (2019) have not used sub-attributes criteria and used 11 attributes with no use of Sanchez’s method. Rahman et al. (2022b) used sub-attributes criteria with 9 attributes. He used Sanchez’s method in one but not in other structure. Ranking of all the structures have been show.

**Table 14:** Comparison analysis of FpNHse-set.

Author’s name	Structure	*NA*	*NP*	Sanchez’s method	Ranking	*UOSA*
Kirişci, Demir & Şi̇mȩek (2021)	S-set	11	6	N/A	${\hat {\Lambda }}_{1}\gt {\hat {\Lambda }}_{2}\gt {\hat {\Lambda }}_{24}\gt {\hat {\Lambda }}_{75}\gt {\hat {\Lambda }}_{25}\gt {\hat {\Lambda }}_{303}$	No
Kirişci (2019)	IFPFS-set	11	6	N/A	${\hat {\Lambda }}_{75}\gt {\hat {\Lambda }}_{24}\gt {\hat {\Lambda }}_{25}\gt {\hat {\Lambda }}_{1}\gt {\hat {\Lambda }}_{2}\gt {\hat {\Lambda }}_{303}$	No
Rahman et al. (2022b)	FPFHS-set	9	6	N/A	${\hat {\Lambda }}_{75}\gt {\hat {\Lambda }}_{2}\gt {\hat {\Lambda }}_{303}\gt {\hat {\Lambda }}_{25}\gt {\hat {\Lambda }}_{25}\gt {\hat {\Lambda }}_{1}$	Yes
Rahman et al. (2022c)	PNHS-set	9	6	Yes	${\hat {\Lambda }}_{24}\gt {\hat {\Lambda }}_{75}\gt {\hat {\Lambda }}_{2}\gt {\hat {\Lambda }}_{1}\gt {\hat {\Lambda }}_{25}\gt {\hat {\Lambda }}_{303}$	Yes
Proposed structure	FpNHse-set	9	6	Yes	${\hat {\Lambda }}_{303}\gt {\hat {\Lambda }}_{24}\gt {\hat {\Lambda }}_{25}\gt {\hat {\Lambda }}_{75}\gt {\hat {\Lambda }}_{2}\gt {\hat {\Lambda }}_{1}$	Yes

In order to evaluate the fluctuation of these score values and rank them, statistical techniques like the Pythagorean mean (arithmetic, geometric, and harmonic means) are used to analyse the sensitivity of the score values of alternatives produced from the approximation of sub-parametric multi-arguments. Table 15’s results demonstrate that even after scores were computed using Pythagorean means, the ordering of alternatives remained constant.

**Table 15:** Sensitivity analysis.

Technique	${\hat {\Lambda }}_{1}$	${\hat {\Lambda }}_{2}$	${\hat {\Lambda }}_{24}$	${\hat {\Lambda }}_{25}$	${\hat {\Lambda }}_{75}$	${\hat {\Lambda }}_{303}$	Ranking
A.M	0.0189	0.0196	0.0253	0.0224	0.0205	0.0327	${\hat {\Lambda }}_{303}\gt {\hat {\Lambda }}_{24}\gt {\hat {\Lambda }}_{25}\gt {\hat {\Lambda }}_{75}\gt {\hat {\Lambda }}_{2}\gt {\hat {\Lambda }}_{1}$
G.M	0.0182	0.0193	0.0249	0.0215	0.0197	0.0323	${\hat {\Lambda }}_{303}\gt {\hat {\Lambda }}_{24}\gt {\hat {\Lambda }}_{25}\gt {\hat {\Lambda }}_{75}\gt {\hat {\Lambda }}_{2}\gt {\hat {\Lambda }}_{1}$
H.M	0.0175	0.0182	0.0245	0.0208	0.0191	0.0320	${\hat {\Lambda }}_{303}\gt {\hat {\Lambda }}_{24}\gt {\hat {\Lambda }}_{25}\gt {\hat {\Lambda }}_{75}\gt {\hat {\Lambda }}_{2}\gt {\hat {\Lambda }}_{1}$
Suggested app.	0.0756	0.0783	0.1015	0.0898	0.0820	0.1308	${\hat {\Lambda }}_{303}\gt {\hat {\Lambda }}_{24}\gt {\hat {\Lambda }}_{25}\gt {\hat {\Lambda }}_{75}\gt {\hat {\Lambda }}_{2}\gt {\hat {\Lambda }}_{1}$

### Benefits of the proposed structure

The suggested algorithmic MADM method outperforms existing methods in the following specific areas and aspects for heart problem analysis using a neutrosophic hypersoft expert set with fuzzy parameterized degree-based settings:

1. **Comprehensive analysis**: To give a thorough examination of heart issues, the method blends a number of techniques, including fuzzy sets, neutrosophic hypersoft sets, and parameterized degree-based settings. This all-encompassing strategy enables the evaluation of numerous factors, such as speculative facts, professional judgements, and personal preferences, leading to a more thorough understanding of heart diseases.
2. **Handling uncertainty and vagueness**: Working with confusing and speculative data is necessary for cardiac condition analysis. Neutrosophic hypersoft sets and fuzzy sets can be used to express and manage data ambiguity and uncertainty. This decreases the impact of erroneous or incomplete information and enables the detection of heart problems with greater accuracy.
3. **Expert knowledge integration**: The methodology includes expert knowledge through the use of expert sets. Expert ideas and perspectives are crucial to an objective analysis of cardiac disorders. The approach takes use of domain experts’ insights to improve decision-making and increase analytical accuracy.
4. **Parameterized degree-based setting**: A versatile framework for accounting for the fuzzy value assigned to various aspects (parameters) or criteria in heart problem analysis is provided by the parameterized degree-based setup. This makes it possible for decision-makers to assign diverse factors relative weights or priority based on their significance, leading to more focused and relevant analysis results.
5. **Algorithmic approach**: Algorithms are used in the methodology, which provides a scientific and structured framework for examining cardiac issues. As a result, it is simpler to deliver reliable results that are repeatable and consistent, ensuring that the analytical process is transparent and open to independent verification.
6. **Applicability to heart problem analysis**: Because the method was established expressly for cardiac condition analysis, it has been modified to address the unique problems and considerations in this area. Since the method incorporates neutrosophic hypersoft sets, fuzzy sets, and parameterized degree-based setting to take into consideration the complexities of cardiac illnesses, it is better suited for accurate and effective analysis.
7. **Classification of parameters**: Through the use of disjoint sets, the suggested strategy successfully divides parameters into their corresponding sub-parametric values. As a result, the results are more accurate and pertinent because each parameter is taken into account in its appropriate context.
8. **Validation through real-world applications**: The Cleveland data set is used to validate the algorithm in real-world settings, specifically in the diagnosis of heart disease. This validation shows how well the model functions in practical decision-making situations.

Overall, the superior performance of the proposed method can be attributed to its ability to manage uncertainty, account for various expert opinions, classify parameters, handle multi-argument approximations, and show successful applications in real-world decision-making scenarios related to the diagnosis of heart disease.

## Conclusions

By approaching the context of novel mathematical structure, *i.e.,* FpNHse-set, the flaws of already developed researches are settled. These flaws may be of different nature like entitlement of multi-argument approximate domain, the gathering of expert’s opinions in terms of neutrosophic values and the provision of arrangement that is capable of assessing the vague nature of attributes and sub attributes. The aggregation operations of FpNHse-set are utilized to establish a decision based system to judge the risk for heart diseases. The proposed framework employs Sanchez’s medical technique to establish a relationship among the patients, medical experts (decision makers) and fuzzy parameterized sub parametric tuples by matrix representation. The attributes and their related sub attributive values are opted from the Cleveland data set so that the genuineness of the proposed framework may be over viewed. The fuzzy parameterized grades for sub parametric tuples are determined by using arithmetical formulation to ease the understanding. The suggested algorithm is consisting of streamline flow of understandable knowledge which leads to easy computational results and thus ranks the patients. Moreover, the flexibility is judged by comparing the suggested framework with already presented researches. In order to seek further extensions of this framework, the structural modifications may be proceeded in future.

## Supplemental Information

10.7717/peerj-cs.1607/supp-1 Supplemental Information 1 Raw Data

10.7717/peerj-cs.1607/supp-2 Supplemental Information 2 Code

## Additional Information and Declarations

### Data Availability

The following information was supplied regarding data availability: The raw data and code are available in the Supplemental Files.

## References

[ref-1] Abbas M, Murtaza G, Smarandache F (2020). Basic operations on hypersoft sets and hypersoft point. Neutrosophic Sets and Systems.

[ref-2] Abdel-Basset M, Gamal A, Manogaran G, Son LH, Long HV (2020). A novel group decision making model based on neutrosophic sets for heart disease diagnosis. Multimedia Tools and Applications.

[ref-3] Al-Quran A, Hassan N (2016). Fuzzy parameterised single valued neutrosophic soft expert set theory and its application in decision making. International Journal of Applied Decision Sciences.

[ref-4] Al-Sharqi F, Ahmad AG, Al-Quran A (2023). Fuzzy parameterized-interval complex neutrosophic soft sets and their applications under uncertainty. Journal of Intelligent & Fuzzy Systems.

[ref-5] Ali MI, Feng F, Liu X, Min WK, Shabir M (2009). On some new operations in soft set theory. Computers & Mathematics with Applications.

[ref-6] Alkhazaleh S, Salleh AR (2011). Soft expert sets. Advances in Decision Sciences.

[ref-7] Alkhazaleh S, Salleh AR (2014). Fuzzy soft expert set and its application. Applied Mathematics.

[ref-8] Atanassov KT, Atanassov KT (1999). Intuitionistic fuzzy sets.

[ref-9] Bashir M, Salleh AR (2012). Fuzzy parameterized soft expert set.

[ref-10] Broumi S, Smarandache F (2015). Intuitionistic fuzzy soft expert sets and its application in decision making. Journal of New Theory.

[ref-11] Cagman N, Enginoglu S (2011). FP-soft set theory and its applications. Annals of Fuzzy Mathematics and Informatics.

[ref-12] Das S, Pradhan SK, Mishra S, Pradhan S, Pattnaik P (2021). Analysis of heart diseases using soft computing technique.

[ref-13] Debnath S (2021). Fuzzy hypersoft sets and its weightage operator for decision making. Journal of Fuzzy Extension and Applications.

[ref-14] Hassan N, Sayed OR, Khalil AM, Ghany MA (2017). Fuzzy soft expert system in prediction of coronary artery disease. International Journal of Fuzzy Systems.

[ref-15] Hazaymeh A, Abdullah I, Balkhi Z, Ibrahim R (2012). Fuzzy parameterized fuzzy soft expert set. Applied Mathematical Sciences.

[ref-16] Ihsan M, Rahman AU, Saeed M (2021a). Fuzzy hypersoft expert set with application in decision making for the best selection of product. Neutrosophic Sets and Systems.

[ref-17] Ihsan M, Rahman AU, Saeed M (2021b). Hypersoft expert set with application in decision making for recruitment process. Neutrosophic Sets and Systems.

[ref-18] Ihsan M, Rahman AU, Saeed M, Khalifa HAE-W (2021). Convexity-cum-concavity on fuzzy soft expert set with certain properties. International Journal of Fuzzy Logic and Intelligent Systems.

[ref-19] Ihsan M, Saeed M, Khan KA, Nosheen A (2023). An algebraic approach to the variants of convexity for soft expert approximate function with intuitionistic fuzzy setting. Journal of Taibah University for Science.

[ref-20] Ihsan M, Saeed M, Rahman AU (2021). A rudimentary approach to develop context for convexity cum concavity on soft expert set with some generalized results. Punjab University Journal of Mathematics.

[ref-21] Ihsan M, Saeed M, Rahman AU (2022). Neutrosophic hypersoft expert set: theory and applications. Neutrosophic Sets and Systems.

[ref-22] Kirişci M, Demir I, Şi̇mşek N (2021). Soft set based new decision-making method with cardiovascular disease application. Sigma: Journal of Engineering & Natural Sciences/Mühendislik Ve Fen Bilimleri Dergisi.

[ref-23] Kirişci M (2019). Comparison of medical decision-making with intuitionistic fuzzy parametrized fuzzy soft set and Riesz summability. New Mathematics and Natural Computation.

[ref-24] Kirişci M (2020a). A case study for medical decision making with the fuzzy soft sets. Afrika Matematika.

[ref-25] Kirişci M (2020b). Medical decision making with respect to the fuzzy soft sets. Journal of Interdisciplinary Mathematics.

[ref-26] Kumar SU, Inbarani HH, Azar AT (2015). Hybrid bijective soft set-neural network for ECG arrhythmia classification. International Journal of Hybrid Intelligent Systems.

[ref-27] Lashari SA, Ibrahim R, Senan N (2017). Medical data classification using similarity measure of fuzzy soft set based distance measure. Journal of Telecommunication, Electronic and Computer Engineering (JTEC).

[ref-28] Long NC, Meesad P, Unger H (2015). A highly accurate firefly based algorithm for heart disease prediction. Expert Systems with Applications.

[ref-29] Maji PK, Biswas R, Roy AR (2003). Soft set theory. Computers & Mathematics with Applications.

[ref-30] Molodtsov D (1999). Soft set theory—first results. Computers & Mathematics with Applications.

[ref-31] Muthukumar P, Krishnan GSS (2016). A similarity measure of intuitionistic fuzzy soft sets and its application in medical diagnosis. Applied Soft Computing.

[ref-32] O’Brien WJ, Formoso CT, Ruben V, London K (2008).

[ref-33] Rahman AU, Saeed M, Alburaikan A, Khalifa HAE-W (2022a). An intelligent multiattribute decision-support framework based on parameterization of neutrosophic hypersoft set. Computational Intelligence and Neuroscience.

[ref-34] Rahman AU, Saeed M, Mohammed MA, Jaber MM, Garcia-Zapirain B (2022b). A novel fuzzy parameterized fuzzy hypersoft set and riesz summability approach based decision support system for diagnosis of heart diseases. Diagnostics.

[ref-35] Rahman AU, Saeed M, Mohammed MA, Krishnamoorthy S, Kadry S, Eid F (2022c). An integrated algorithmic MADM approach for heart diseases’ diagnosis based on neutrosophic hypersoft set with possibility degree-based setting. Life.

[ref-36] Rahman AU, Saeed M, Smarandache F (2020). Convex and concave hypersoft sets with some properties. Neutrosophic Sets and Systems.

[ref-37] Rahman AU, Saeed M, Smarandache F, Ahmad MR (2020). Development of hybrids of hypersoft set with complex fuzzy set, complex intuitionistic fuzzy set and complex neutrosophic set. Neutrosophic Sets and Systems.

[ref-38] Saeed M, Ahsan M, Saeed MH, Mehmood A, Abdeljawad T (2021a). An application of neutrosophic hypersoft mapping to diagnose hepatitis and propose appropriate treatment. IEEE Access.

[ref-39] Saeed M, Ahsan M, Ur Rahman A, Saeed MH, Mehmood A (2021b). An application of neutrosophic hypersoft mapping to diagnose brain tumor and propose appropriate treatment. Journal of Intelligent & Fuzzy Systems.

[ref-40] Saeed M, Rahman AU, Ahsan M, Smarandache F (2022). Theory of hypersoft sets: axiomatic properties, aggregation operations, relations, functions and matrices. Neutrosophic Sets and Systems.

[ref-41] Sanchez E (1979). Inverses of fuzzy relations. Application to possibility distributions and medical diagnosis. Fuzzy Sets and Systems.

[ref-42] Sanz JA, Galar M, Jurio A, Brugos A, Pagola M, Bustince H (2014). Medical diagnosis of cardiovascular diseases using an interval-valued fuzzy rule-based classification system. Applied Soft Computing.

[ref-43] Saqlain M, Saeed M, Ahmad MR, Smarandache F (2019). Generalization of TOPSIS for neutrosophic hypersoft set using accuracy function and its application. Neutrosophic Sets and Systems.

[ref-44] Selvachandran G, Salleh AR (2016). Fuzzy parameterized intuitionistic fuzzy soft expert set theory and its application in decision making. International Journal of Soft Computing.

[ref-45] Smarandache F (2006). Neutrosophic set-a generalization of the intuitionistic fuzzy set.

[ref-46] Smarandache F (2018). Extension of soft set to hypersoft set, and then to plithogenic hypersoft set. Neutrosophic Sets and Systems.

[ref-47] UCI Machine Learning Repository (2010). https://archive.ics.uci.edu/ml/datasets/heart+Disease.

[ref-48] Yılmaz Ş, Eraslan S (2012). Fuzzy parametrized fuzzy soft set and Riesz summability applying to a decision making problem. Gaziosmanpaşa Bilimsel Araa̧trma Dergisi.

[ref-49] Yolcu A, Smarandache F, Öztürk TY (2021). Intuitionistic fuzzy hypersoft sets. Communications Faculty of Sciences University of Ankara Series A1 Mathematics and Statistics.

[ref-50] Zadeh LA (1965). Fuzzy sets. Information and Control.

