# Supplementary material for: An algorithmic multiple attribute decision-making method for heart problem analysis under neutrosophic hypersoft expert set with fuzzy parameterized degree-based setting
Source: PeerJ Comput Sci. 2023 Dec 4;9:e1607. doi: 10.7717/peerj-cs.1607 (PMC10703036; doi:10.7717/peerj-cs.1607)
Supplement: Supplemental Information 1 [file peerj-cs-09-1607-s001.docx]

Raw Data

**Manuscript Title: An algorithmic MADM method for heart problem analysis under neutrosophic hypersoft expert set with fuzzy parameterized degree-based setting**

Following are the sources for the raw considered in the above-mentioned manuscript:

1. **Parameters and their respective sub parametric values**

The parameters and their respective sub parametric values are taken from the **“Cleveland data set for heart disease”** which is freely available online at <http://archive.ics.uci.edu/ml/datasets/Heart+Disease> by UCI Machine Learning Repository (<http://archive.ics.uci.edu/ml>). The citation and acknowledgment policies of the above mentioned provider have been followed by the authors in the submitted manuscript.

The complete information of the above mentioned data set is provided below:

| **Data Set Characteristics:** | Multivariate | **Number of Instances:** | 303 | **Area:** | Life |
| --- | --- | --- | --- | --- | --- |
| **Attribute Characteristics:** | Categorical, Integer, Real | **Number of Attributes:** | 75 | **Date Donated** | 1988-07-01 |
| **Associated Tasks:** | Classification | **Missing Values?** | Yes | **Number of Web Hits:** | 2145694 |

Data Set Information:

This database contains 76 attributes, but all published experiments refer to using a subset of 14 of them. In particular, the Cleveland database is the only one that has been used by ML researchers to this date. The "goal" field refers to the presence of heart disease in the patient. It is integer valued from 0 (no presence) to 4. Experiments with the Cleveland database have concentrated on simply attempting to distinguish presence (values 1,2,3,4) from absence (value 0). The names and social security numbers of the patients were recently removed from the database, replaced with dummy values. One file has been "processed", that one containing the Cleveland database. All four unprocessed files also exist in this directory. To see Test Costs (donated by Peter Turney), please see the folder "Costs"

1. **Attribute Information:**

Only 14 attributes used:

1. #3 (age)

2. #4 (sex)

3. #9 (cp)

4. #10 (trestbps)

5. #12 (chol)

6. #16 (fbs)

7. #19 (restecg)

8. #32 (thalach)

9. #38 (exang)

10. #40 (oldpeak)

11. #41 (slope)

12. #44 (ca)

13. #51 (thal)

14. #58 (num) (the predicted attribute)

1. **Complete attribute documentation:**
2. id: patient identification number
3. ccf: social security number (I replaced this with a dummy value of 0)
4. age: age in years
5. sex: sex (1 = male; 0 = female)
6. painloc: chest pain location (1 = substernal; 0 = otherwise)
7. painexer (1 = provoked by exertion; 0 = otherwise)
8. relrest (1 = relieved after rest; 0 = otherwise)
9. pncaden (sum of 5, 6, and 7)
10. cp: chest pain type

-- Value 1: typical angina

-- Value 2: atypical angina

-- Value 3: non-anginal pain

-- Value 4: asymptomatic

1. trestbps: resting blood pressure (in mm Hg on admission to the hospital)
2. htn
3. chol: serum cholestoral in mg/dl
4. smoke: I believe this is 1 = yes; 0 = no (is or is not a smoker)
5. cigs (cigarettes per day)
6. years (number of years as a smoker)
7. fbs: (fasting blood sugar > 120 mg/dl) (1 = true; 0 = false)
8. dm (1 = history of diabetes; 0 = no such history)
9. famhist: family history of coronary artery disease (1 = yes; 0 = no)
10. restecg: resting electrocardiographic results

-- Value 0: normal

-- Value 1: having ST-T wave abnormality (T wave inversions and/or ST elevation or depression of > 0.05 mV)

-- Value 2: showing probable or definite left ventricular hypertrophy by Estes' criteria

1. ekgmo (month of exercise ECG reading)
2. ekgday(day of exercise ECG reading)
3. ekgyr (year of exercise ECG reading)
4. dig (digitalis used furing exercise ECG: 1 = yes; 0 = no)
5. prop (Beta blocker used during exercise ECG: 1 = yes; 0 = no)
6. nitr (nitrates used during exercise ECG: 1 = yes; 0 = no)
7. pro (calcium channel blocker used during exercise ECG: 1 = yes; 0 = no)
8. diuretic (diuretic used used during exercise ECG: 1 = yes; 0 = no)
9. proto: exercise protocol

1 = Bruce

2 = Kottus

3 = McHenry

4 = fast Balke

5 = Balke

6 = Noughton

7 = bike 150 kpa min/min (Not sure if "kpa min/min" is what was written!)

8 = bike 125 kpa min/min

9 = bike 100 kpa min/min

10 = bike 75 kpa min/min

11 = bike 50 kpa min/min

12 = arm ergometer

1. thaldur: duration of exercise test in minutes
2. thaltime: time when ST measure depression was noted
3. met: mets achieved
4. thalach: maximum heart rate achieved
5. thalrest: resting heart rate
6. tpeakbps: peak exercise blood pressure (first of 2 parts)
7. tpeakbpd: peak exercise blood pressure (second of 2 parts)
8. Dummy
9. trestbpd: resting blood pressure
10. exang: exercise induced angina (1 = yes; 0 = no)
11. xhypo: (1 = yes; 0 = no)
12. oldpeak = ST depression induced by exercise relative to rest
13. slope: the slope of the peak exercise ST segment

-- Value 1: upsloping

-- Value 2: flat

-- Value 3: downsloping

1. rldv5: height at rest
2. rldv5e: height at peak exercise
3. ca: number of major vessels (0-3) colored by flourosopy
4. restckm: irrelevant
5. exerckm: irrelevant
6. restef: rest raidonuclid (sp?) ejection fraction
7. restwm: rest wall (sp?) motion abnormality

0 = none

1 = mild or moderate

2 = moderate or severe

3 = akinesis or dyskmem (sp?)

1. exeref: exercise radinalid (sp?) ejection fraction
2. exerwm: exercise wall (sp?) motion
3. thal: 3 = normal; 6 = fixed defect; 7 = reversable defect
4. thalsev: not used
5. thalpul: not used
6. earlobe: not used
7. cmo: month of cardiac cath (sp?) (perhaps "call")
8. cday: day of cardiac cath (sp?)
9. cyr: year of cardiac cath (sp?)
10. num: diagnosis of heart disease (angiographic disease status)

-- Value 0: < 50% diameter narrowing

-- Value 1: > 50% diameter narrowing

(in any major vessel: attributes 59 through 68 are vessels)

1. Lmt
2. Ladprox
3. Laddist
4. Diag
5. Cxmain
6. Ramus
7. om1
8. om2
9. Rcaprox
10. Rcadist
11. lvx1: not used
12. lvx2: not used
13. lvx3: not used
14. lvx4: not used
15. lvf: not used
16. cathef: not used
17. junk: not used
18. name: last name of patient (I replaced this with the dummy string "name")
19. **Softwares**
20. Microsoft Excel 2010
21. Microsoft Word 2010
22. WinEdt version Build: 20121130 (v. 7.0)
23. MathType version 6.9
