# Supplementary material for: An algorithmic multiple attribute decision-making method for heart problem analysis under neutrosophic hypersoft expert set with fuzzy parameterized degree-based setting
Source: PeerJ Comput Sci. 2023 Dec 4;9:e1607. doi: 10.7717/peerj-cs.1607 (PMC10703036; doi:10.7717/peerj-cs.1607)
Supplement: Supplemental Information 2 [file peerj-cs-09-1607-s002.docx]

**Code**

**Manuscript Title: An algorithmic MADM method for heart problem analysis under neutrosophic hypersoft expert set with fuzzy parameterized degree-based setting**

In the above-mentioned manuscript, the authors have not used any machine learning tools or computer languages based softwares therefore there is no coding involved in the manuscript to design the algorithm. However, the methodological steps, without coding indices, in general template are being presented below that can be executed by any machine learning tool after transformation in codes:

1. Taking expert's set
2. Expert opinions $\left\{0=disagree,1=agree\right\}$
3. Set of parameters
4. Sort q parameters into distinct sets with parametric values:

,

,

,

------------------------------,

------------------------------,

,

1. Calculate
2. Select with $r\leq q$.
3. Construction of FpNHse-sets and their representation in matrices form and respectively.
4. Determine having entries as $\frac{\left|t-f-i\right|}{3}$ and same as for
5. Determine the matrix by adding the 3rd and 4rth matrices.
6. Determine the scores by finding rows addition of 5th matrix.
7. Select the maximum score for decision.
